# *AutoDockFR*: Advances in Protein-Ligand Docking with Explicitly Specified Binding Site Flexibility

**DOI:** 10.1371/journal.pcbi.1004586

**Published:** 2015-12-02

**Authors:** Pradeep Anand Ravindranath, Stefano Forli, David S. Goodsell, Arthur J. Olson, Michel F. Sanner

**Affiliations:** Department of Integrative Structural and Computational Biology, The Scripps Research Institute, La Jolla, California, United States of America; Wake Forest University, UNITED STATES

## Abstract

Automated docking of drug-like molecules into receptors is an essential tool in structure-based drug design. While modeling receptor flexibility is important for correctly predicting ligand binding, it still remains challenging. This work focuses on an approach in which receptor flexibility is modeled by explicitly specifying a set of receptor side-chains a-priori. The challenges of this approach include the: 1) exponential growth of the search space, demanding more efficient search methods; and 2) increased number of false positives, calling for scoring functions tailored for flexible receptor docking. We present *AutoDockFR*–*AutoDock* for Flexible Receptors (*ADFR*), a new docking engine based on the *AutoDock4* scoring function, which addresses the aforementioned challenges with a new Genetic Algorithm (GA) and customized scoring function. We validate *ADFR* using the Astex Diverse Set, demonstrating an increase in efficiency and reliability of its GA over the one implemented in *AutoDock4*. We demonstrate greatly increased success rates when cross-docking ligands into *apo* receptors that require side-chain conformational changes for ligand binding. These cross-docking experiments are based on two datasets: 1) SEQ17 –a receptor diversity set containing 17 pairs of *apo-holo* structures; and 2) CDK2 –a ligand diversity set composed of one CDK2 *apo* structure and 52 known bound inhibitors. We show that, when cross-docking ligands into the *apo* conformation of the receptors with up to 14 flexible side-chains, *ADFR* reports more correctly cross-docked ligands than *AutoDock Vina* on both datasets with solutions found for 70.6% vs. 35.3% systems on SEQ17, and 76.9% vs. 61.5% on CDK2. *ADFR* also outperforms *AutoDock Vina* in number of top ranking solutions on both datasets. Furthermore, we show that correctly docked CDK2 complexes re-create on average 79.8% of all pairwise atomic interactions between the ligand and moving receptor atoms in the *holo* complexes. Finally, we show that down-weighting the receptor internal energy improves the ranking of correctly docked poses and that runtime for *AutoDockFR* scales linearly when side-chain flexibility is added.

## Author Summary

Docking programs are widely used to identify drug-like molecules interacting with a given receptor to inhibit its function. Although receptors are known to change conformation upon ligand binding, most docking programs model small molecules as flexible while modeling receptors as rigid, thus limiting the range of therapeutic targets for which docking can be applied. Here we introduce a new docking program, *AutoDockFR*, which simulates partial receptor flexibility by allowing a large number of explicitly specified receptor side-chains to explore their conformational space, while searching for energetically favorable binding poses for a given ligand. We show that we achieve higher docking success rates by including receptor flexibility in the binding site of receptor conformations that are experimentally determined without the ligand present (i.e. *apo* conformations). Previous approaches based on the a-priori and explicit specification of the part of the receptor to be considered flexible, have so far been limited to a small number of flexible protein side-chains (2–5), thus requiring prior knowledge of receptor side-chains undergoing conformational change upon binding of a given ligand. The demonstrated ability of *AutoDockFR* in identifying correct solutions for problems with up to 14 flexible receptor side-chains lessens this requirement.

“This is a *PLOS Computational Biology* Methods paper”

## Introduction

Structure-based computational drug design is an essential tool in computational medicinal chemistry [1–3]. Docking is used for optimizing known drugs and for identifying novel binders by predicting their binding mode and affinity [4, 5]. While the exploration of the ligand conformational space during the docking procedure is common, modeling receptor flexibility upon ligand binding still remains a major challenge because of the computational resources required [6]. Recent reviews provide an excellent and detailed analysis of state-of-the-art techniques for modeling receptor flexibility in structure-based drug design [7, 8]. In summary, the motions induced in receptors upon ligand binding range from small local adjustments to large re-arrangements [9]. Modeling the receptor as fully flexible during the docking calculation is too expensive computationally because of the large number of degrees of freedom to explore during the search [10]. Instead a number of computationally feasible approximations have been proposed that can broadly be classified into the following three categories: 1) methods altering interaction potentials, where repulsive potentials between ligand and receptor atoms are attenuated [11] or grids representing these potentials are deformed [12], or a consensus potential is created to represent various conformations of the receptor [13]; 2) ensemble docking methods [13, 14], using a discrete set of receptor conformations; and 3) induced fit methods, where changes in receptor conformation are explored during the docking [15–24]. Some approaches may fall into multiple categories depending on the classification criteria. Potential altering approaches are computationally inexpensive; however the range of motions they can account for is rather limited. The elastic deformation of affinity grids has been shown to be a computationally effective way to increase the accuracy of cross-docking ligands into non-native structure. However, the authors observed that this approach failed on a case where a large receptor side-chain conformational change is needed for the ligand to bind. The ensemble docking approach does not require any modification to existing docking codes, is embarrassingly parallel, and has been used successfully in the design of an HIV reverse transcriptase inhibitor [25]. The success rate of this method depends on the presence of a suitable receptor conformation for the ligand being docked. This limitation is somewhat attenuated in approaches that use receptor conformations to define receptor fragments that are combined during the docking procedure, thus exploring a larger subset of the receptor conformational space [15, 16]. Induced fit methods vary in their strategies for accounting for receptor and ligand flexibility. Some methods rely on pre-computed, low energy ligand conformers which are placed into the receptor structure, and either re-pack the receptor side-chains around the docked ligand, or adjust the receptor and the ligand conformations to resolve clashes. These techniques do not require the a-priori specification of the receptor side-chains to be made flexible and can potentially modify the conformation of a large number of binding-site side-chains. SLIDE [17] resolves the clashes by minimal rotations [18] and mean-field optimization of a simplified scoring function, making it efficient for virtual screening studies. In addition to modeling the motions of receptor side-chains, Rosetta Ligand can also induce changes in the backbone conformation [26], but this approach is computationally expensive. Other methods [15, 16, 20–24, 27] rely on the explicit and a-priori specification of the parts of the receptor to be made flexible. These methods explore a solution space spanning all possible ligand rotations and translations, and all possible conformations of both the ligand and the flexible parts the receptor. *ADFR* falls into this category, which we refer to “*explicit methods”* as they require the explicit specification of the flexible parts of the receptor prior to docking. While these approaches have mostly focused on receptor side-chain motions, some of them also include limited backbone motion [15, 16, 23, 27]. The main challenges of explicit methods include: 1) the difficulty of finding the global minimum in solution-spaces that grow exponentially with the number of degrees of freedom added by the receptor; and 2) the increased number of false positives arising from the evaluation of more potential solutions, using scoring functions with inherent approximations and defects as underlined in [28]. Because of these limitations, reports of successful usage of these programs have been limited to docking studies with relatively small numbers of flexible receptor side-chains, typically 2–5, putting the burden of selecting the side-chains that will move on the user.

*AutoDock* is a widely used docking program that allows the specification of flexible side-chains. However, its hardcoded limit of 32 rotatable bonds is easily exceeded when receptor side-chains are made flexible. Moreover, the Genetic Algorithm (GA) implemented in *AutoDock* does not perform well for docking complexes with more than ~20 rotatable bonds. Here, we present a new docking engine–*AutoDockFR*: *AutoDock* for Flexible Receptors (*ADFR)* - implementing a new genetic algorithm. We demonstrate its application to the high-dimensional solution spaces corresponding to docking a fully flexible ligand into a receptor with up to 14 explicitly specified, flexible side-chains. While *ADFR* is designed to allow the inclusion of a wide variety of receptor motions, this work focuses on receptor motion occurring in receptor side-chains with minor backbone motion. The previously developed Flexibility Tree (FT) data structure supports the encoding of a wide variety of hierarchically nested molecular motions [29] and was first used in our earlier docking software *FLIPDock* [23]. *AutoDockFR* supersedes *FLIPDock* and introduces a new and more efficient Genetic Algorithm (GA), as well as a new motion descriptor for the Flexibility Tree optimized for representing flexible receptor side-chains. The new GA developed for *ADFR* introduces the concept of clustering of the ensemble of solutions optimized by the GA (i.e. the population). Clustering enables maintaining diversity in the population and the implementation of an efficient termination criterion. This new GA also implements a new strategy for minimizing solutions during the GA optimization. In this paper, we provide an overview of the algorithm and describe the key concepts supporting the efficiency of the new GA. We validate the implementation of the *AutoDock4* scoring function [30] and quantify its improvement in efficiency over the one implemented in *AutoDock* by re-docking ligands of the Astex Diverse set into their native rigid receptors. Next, we demonstrate the ability of *ADFR* to cross-dock flexible ligands into flexible *apo* receptors using two datasets, one emphasizing receptor diversity and the other on ligand diversity. The first dataset (SEQ17) comprises 17 diverse *apo*-*holo* receptor pairs. These 17 systems were selected to represent a wide range of receptors and present at least one severe clash between a ligand atom and a receptor side-chain in its *apo* conformation. We show that *ADFR* significantly increases the docking success rate over *AutoDock Vina* when cross-docking each ligand into the *apo* conformation of its receptor, with up to 14 flexible receptor side-chains. The second dataset comprises an *apo* conformation of the Cyclin Dependent Kinase receptor (CDK2) and 52 ligands from *holo* complexes of this receptor. The 52 ligands are docked into the *apo* conformation of the receptor with a number of flexible receptor side-chains varying from 0 to 12. We show that increasing the number of flexible side-chains increases the docking success rate, and that *ADFR* achieves better success rates than *AutoDock Vina* with a linear scaling in run time when increasing the number of flexible receptor side-chains. For the CDK2 cross-docking experiment we also provide a detailed analysis of conformational changes induced by the ligands in the twelve side-chains made flexible in the *apo* conformation. We show that, in the docked complexes, the receptor side-chains move to re-create on average 79.8% of the atomic pairwise interactions observed in the *holo* complex. Finally, we show that in both cross-docking experiments, down weighting the contribution of the receptor internal energy in the score increases the ranking of correctly docked solutions.

## Methods

### Algorithm overview

The three main components of docking programs are: the representation (i.e. the encoding of the docking problem as a set of variables to be optimized), the scoring function for which these variable are optimized, and the search method. *ADFR* encodes the docking problem into a list of variables describing a docking solution and optimizes it for the *AutoDock4* force field using a Genetic Algorithm (GA) combined with a Solis-Wets local search [31]. The source code of the program is available online along with binaries and all input files for reproducing calculations reported in this paper [http://adfr.scripps.edu/].

### Representation

**Fig 1:**
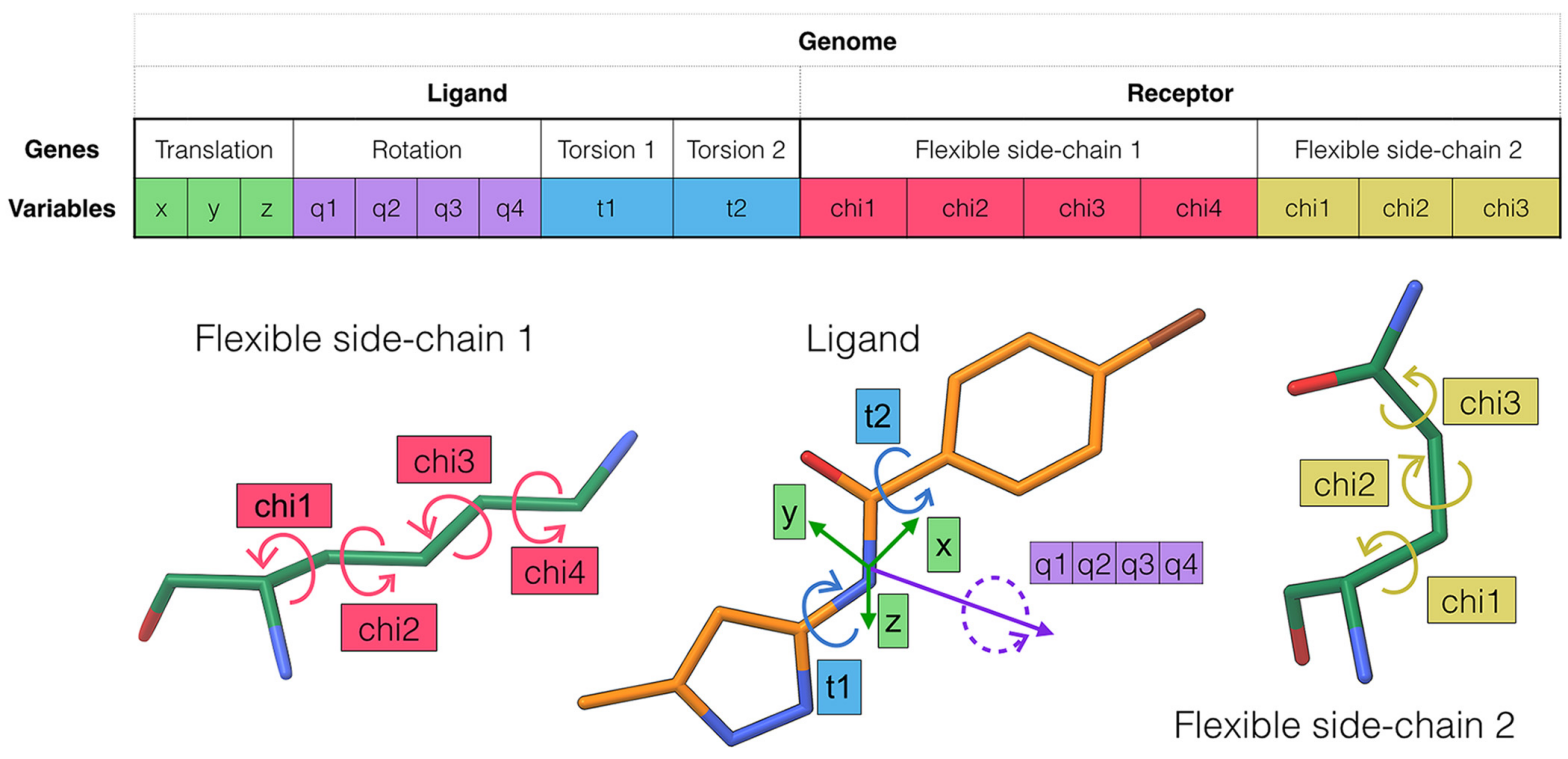
Genome used by *ADFR* to encode the docking of a flexible ligand into a receptor with two flexible side-chains. This figure illustrates a genome optimized by the GA implemented in *ADFR* for solving the problem of docking a flexible ligand with two rotatable bonds into a receptor with two flexible side-chains. The genome is the set of variables to optimize. A given set of values for these variables constitutes a docking solution also called an individual. Variables are grouped into the following genes: the ligand translation (3 values: x, y, z), rotation (4 values: quaternion), and conformation (1 torsion angle per ligand rotatable bond), and the receptor conformation (χ angles for each flexible receptor side-chain).

In *ADFR*, the problem of docking a flexible ligand into a receptor with flexible side-chains is encoded as a set of variables called a genome, and representing the degrees of freedom associated with: (i) the ligand orientation (rotation and translation); (ii) the ligand conformation; and (iii) the receptor conformation; (Fig 1). In our approach, the ligand translation adds three variables to the genome. The rotation of the ligand is described by a quaternion[32], which adds four variables corresponding to a quaternion. Quaternion representation is used over Euler angles to avoid gimbal lock singularities and for stable interpolations of rotations. The ligand conformation is encoded as torsion-angle values for rotatable bonds in the ligand. Hence, a ligand with two rotatable bonds will add two variables to the genome for its conformation. The receptor conformational changes are currently limited to side-chain motions. Each flexible side-chain adds its list of χ angles to the genome. For instance, a lysine will add four variables to the genome when made flexible. Fig 1 shows an example of a genome for a ligand with two rotatable bonds and a receptor with two flexible side-chains. Related variables in the genome are called genes and are implemented as programmatic objects. For instance the three variables corresponding to the translation of the ligand are grouped in a translation gene object. This object-oriented approach enables the implementation of gene-specific operations for the initialization, randomization, perturbation, and mutation of the gene values. For instance the initialization operator of the ligand translation gene object randomly picks a translation from a pre-defined set, while the initialization operator of flexible receptor side-chains gene object initializes the χ angles with the angles obtained from the input conformation of the receptor. Likewise, the mutation of the translation genes modifies the x,y,z values of the gene using a Gaussian distribution centered on its current values, while the mutation operator of a flexible receptor side-chain object randomly selects a set of rotameric angles (with deviations) from the rotamer library. This object-oriented architecture is instrumental for the focused sampling of various dimensions of the search space (see below), which is one of the key features for successfully searching large solutions spaces. A given set of values for the variables in the genome (*i*.*e*. the genotype) corresponds to a docking solution for which the coordinates of the receptor and ligand atoms (*i*.*e*. phenotype) can be calculated and used to compute the value of the scoring function for this solution. In *ADFR* the genome is assembled dynamically at runtime from the description of molecular flexibility provided in the input files. The ligand is specified using the *AutoDock* file format (*i*.*e*. PDBQT), which describes ligand rotatable bonds. The receptor side-chains to be made flexible are specified in the docking settings file using residue names (i.e. residue type and number). The ligand translation is limited to a set of possible values called translational points (see below), which are stored in a file specified in the docking settings file.

Below we describe the scoring function and the GA implemented in *AutoDockFR*, followed by a description of focused sampling techniques that support the GA performance.

### Scoring function

The *AutoDock* energy function [30] (Eq 1) is a weighted sum of terms representing van der Waals, hydrogen bond, electrostatic, and desolvation contributions, which are calculated between pairs of atoms.

$$E=w_{vdW}\;\sum_{i,j}\left(\frac{A_{i,j}}{r_{i,j}^{12}}-\;\frac{B_{i,j}}{r_{i,j}^{6}}\right)+\;w_{hbond}\;\sum_{i,j}\;E\left(t\right)\left(\frac{C_{i,j}}{r_{i,j}^{12}}-\;\frac{D_{i,j}}{r_{i,j}^{10}}\right)+w_{elec}\;\sum_{i,j}\left(\frac{q_{i}q_{j}}{{\epsilon \;r}_{i,j}.r_{i,j}}\right)+\;w_{sol}\;\sum_{i,j}\left(S_{i}V_{j}+\;S_{j}V_{i}\right)e^{\left(\frac{{-r}_{ij}^{2}}{{2\sigma }^{2}}\right)}$$ (Eq. 1)

The *ADFR* score (Eq 2) uses this energy function to independently score the interactions between the following three groups of atoms: Ligand atoms (L), Rigid Receptor atoms (RR) and Flexible Receptor atoms (FR). The total score is the sum of these interaction terms:

$$S_{ADFR}=\;E_{L-L}+E_{L-RR}+E_{L-FR}+E_{FR-FR}+E_{FR-RR}$$ (Eq. 2)

In the case of a rigid receptor, only the first two terms (i.e., *E*_*L-L*_ or ligand intra-molecular and *E*_*L-RR*_ or ligand-rigid receptor inter-molecular interactions) are considered. The additional terms (*E*_*L-FR*_, *E*_*FR-FR*_, *E*_*FR-RR*_) are automatically included in the scoring functions when receptor atoms are made flexible. A weight can be assigned to each term of the scoring function. Similarly to *AutoDock*, *ADFR* uses affinity maps to represent interactions between ligand or flexible receptor atoms and rigid receptor atoms; hence the E_L-RR_ and E_FR-RR_ terms are efficiently obtained by interpolating values in affinity maps. The remaining terms (E_L-L,_ E_L-FR,_ E_FR-FR_) are computed using explicit atom pairs for every non-bonded pair of atoms excluding 1–3 interactions, and 1–4 interactions not mediated by a rotatable bond.

**Fig 2:**
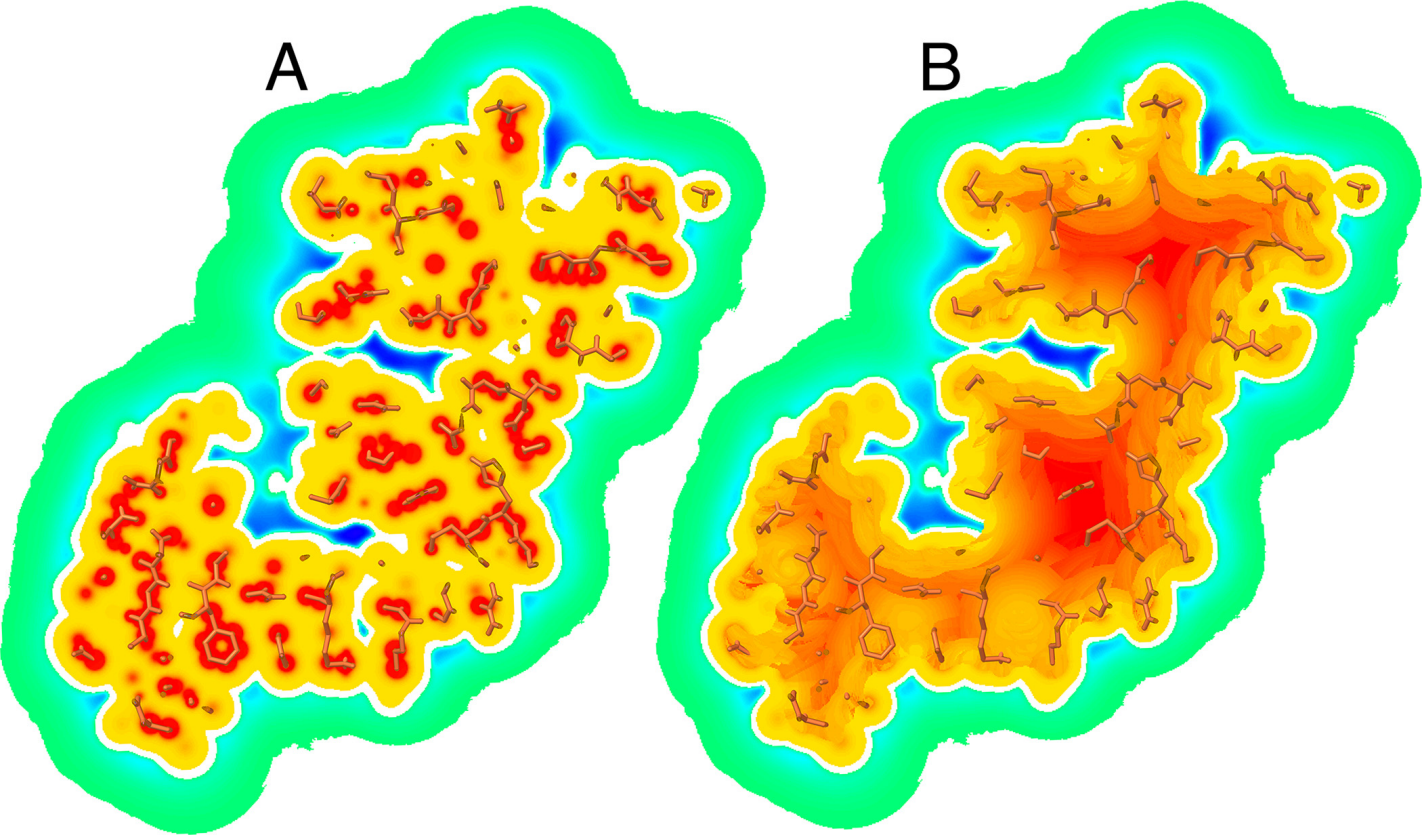
Affinity maps processing. A) A cross-section of the *AutoDock* carbon affinity map. B) The same cross-section after processing the map to create a gradient inside the protein. Besides creating a potential gradient inside the receptor, this processing also removes the local minima inside the receptor volume. The color gradient outside the protein surface indicates favorable interactions going from weak (green) to strong (blue). Inside the protein surface the color gradient indicates unfavorable interactions going from low (yellow) to highly unfavorable (red).

Affinity maps are regular 3D grids defined on a box aligned with the Cartesian axis. This box defines the space that ligand atoms can occupy. Affinity maps are computed prior to docking using *AutoGrid* from the *AutoDockTools* suite [20] with a default grid map spacing of 0.375Å. Affinity values calculated for grid points inside the protein present dramatic fluctuations with the highest values centered on receptor atoms, and the potential falling off rapidly around the atom centers (Fig 2A). We designed a map post-processing protocol, which replaces the potential on grid points inside the receptor with a repulsive potential that provides a gradient pointing toward ligand binding surface (Fig 2B). While this figure shows an example of an open pocket, the same protocol works for a buried cavity. This protocol produces maps that facilitate the search by providing a gradient for resolving clashes and by removing buried favorable cavities too small to accommodate a ligand e.g., trapped water cavities.

### Genetic Algorithm (GA)

**Fig 3:**
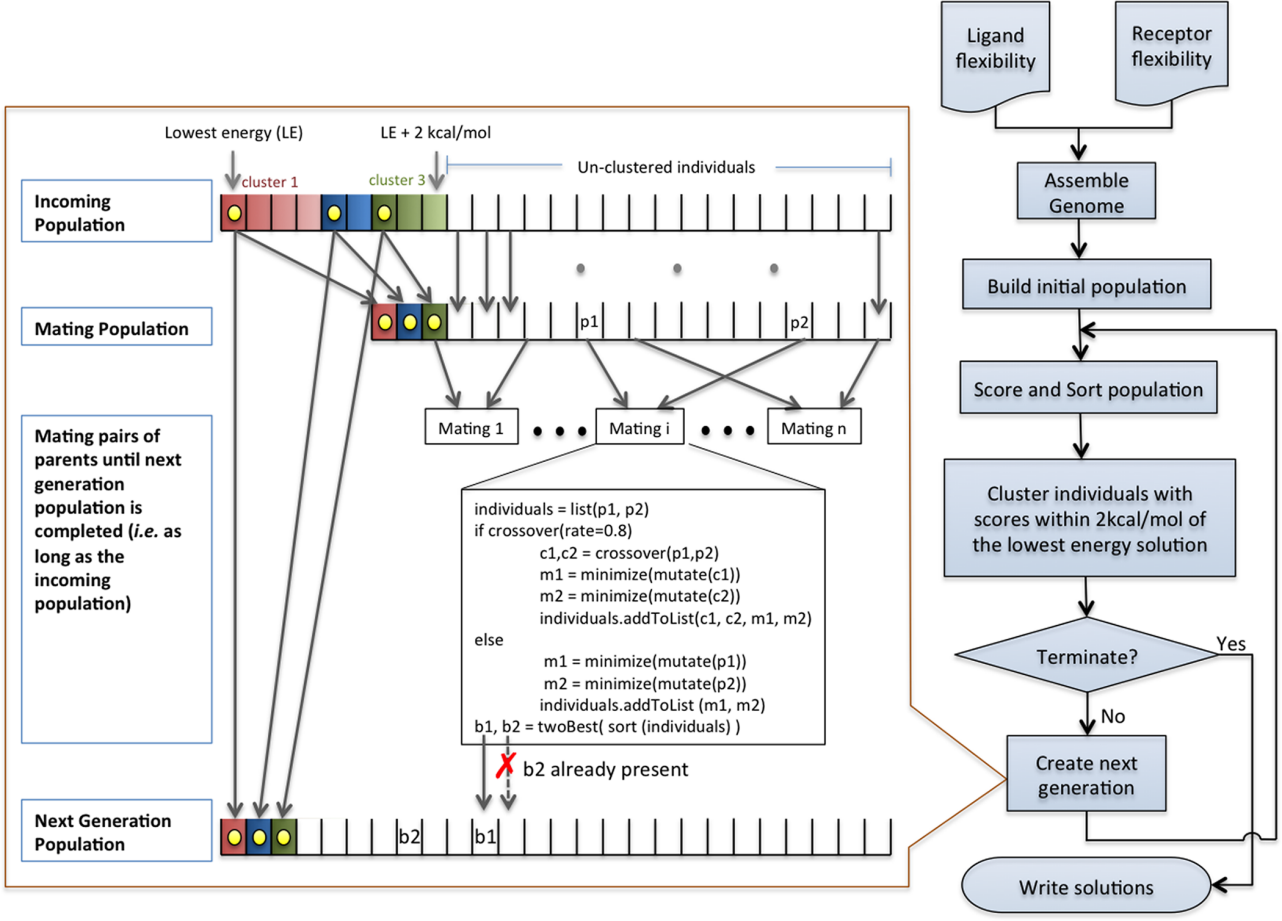
Overall flowchart of ADFR. The flexibility information of the ligand (i.e. rotatable bonds) and receptor (i.e. flexible side-chains) is used to assemble the genome from which an initial list of solutions (i.e. population) is created. The population is scored, sorted, and top-ranking solutions are clustered. The GA seeds the next generation with the best solution of each cluster and completes it by crossing-over, mutating, and minimizing individuals from the mating population. The optimization stops when one of the termination criteria (maximum number of generations or evaluations) is reached or the search converges, at which point the solutions within 1 kcal/mol of the best solution are written out.

The overall workflow of *ADFR* is depicted in Fig 3. The ligand and receptor flexibility description is first used to assemble a list of variables (genome) encoding the flexible ligand—flexible receptor docking problem. The initial population is then generated by creating a list of initial solutions in which each solution (i.e. an individual) is a list of values, one for each variable in the genome. Initial ligand translations are randomly selected from translational points (see below); rotations are initialized using random quaternions; ligand torsions are set to random angles; and finally, flexible side chains are initialized with χ angles from the input conformation of the receptor. The size of the population can be specified by the user or can be inferred by *ADFR*. Once the initial population has been generated, the GA will optimize it by creating successive generations as follows. First, the population is sorted and the top-ranking individuals (i.e. within 2kcal/mol of the lowest energy solution) are clustered. Clustering of solutions is used to remove duplicate solutions from the population, thus ensuring diversity. It also supports the implementation of adaptive elitism by automatically adding to the next generation the best solution from each cluster. The use of clustering in our implementation leads to the simultaneous exploration and optimization of multiple minima during the search. Clustering also enables the implementation of an efficient termination criterion described below. The clustering is performed by using the lowest energy, not yet clustered solution as a cluster seed, and adding to the cluster all solutions with RMSD less than 2Å (for ligand atoms) with respect to the cluster seed. The procedure is repeated until all solutions to be clustered belong to a cluster. Next a mating population, containing the best individual of each cluster along with all un-clustered individuals is created. The best individual from each cluster is automatically copied into the next generation (adaptive elitism). The GA then selects parents to crossover, mutate, and minimize to generate offspring, which compete with their parents to be added to the next generation population. The probability of an individual to have offspring is proportional to its score. A pair of parents selected for mating is crossed-over 80% of the time and the resulting offspring are mutated and minimized. In the case, where no crossover takes place (20% of the time), the two parents are mutated and minimized to obtain offspring. Details of the implementation of crossover, mutation, and minimization are provided in Supporting Information [S1 Text]. All created individuals undergo a quick minimization step. If the minimized individual has a score that is better than the reference score (best score seen so far), it undergoes a more aggressive minimization and its score becomes the reference score. The best two individuals identified during this mating procedure are added to the next generation if they are not already present in that population. Once the population for the next generation is complete (i.e. its size reaches the size of the incoming population) it becomes the incoming population for the next generation in the GA optimization loop.

If the clusters remain unchanged (i.e. the same number of clusters and the energy of the best solution of each cluster remains the same) for three consecutive generations, the entire population is submitted to an aggressive minimization. If the clusters remain unchanged for five consecutive generations the search is considered to have converged and the optimizations stops. The optimization also stops if user-specified limits such as the maximum number of generations or maximum number of scoring function evaluations are reached. After the optimization stops, the solutions within 1kcal/mol of the best solution are clustered and the best solution from each cluster is written to a file.

By default, an *ADFR* docking experiment performs 50 independent GA evolutions, each producing one solution. These solutions are then clustered to remove duplicated solutions and the best scoring individual from each cluster is reported, resulting in a ranked list of solutions for the docking.

### Focused sampling of the solution space

The solution space explored during an *ADFR* calculation is very large and reducing the extent of any of the variables in the genome facilitates the search. In *ADFR* we apply this principle by reducing the sampling of the ligand’s translation to a sub-space of translations more likely to yield good docking poses, thus eliminating ligand translations known to place it either inside the receptor, or too close or too far from the receptor. Likewise, so called “soft-rotamers” (see below) allow *ADFR* to sample receptor side-chain conformations resembling the ones observed in crystal structures more frequently.

#### Translational points

**Fig 4:**
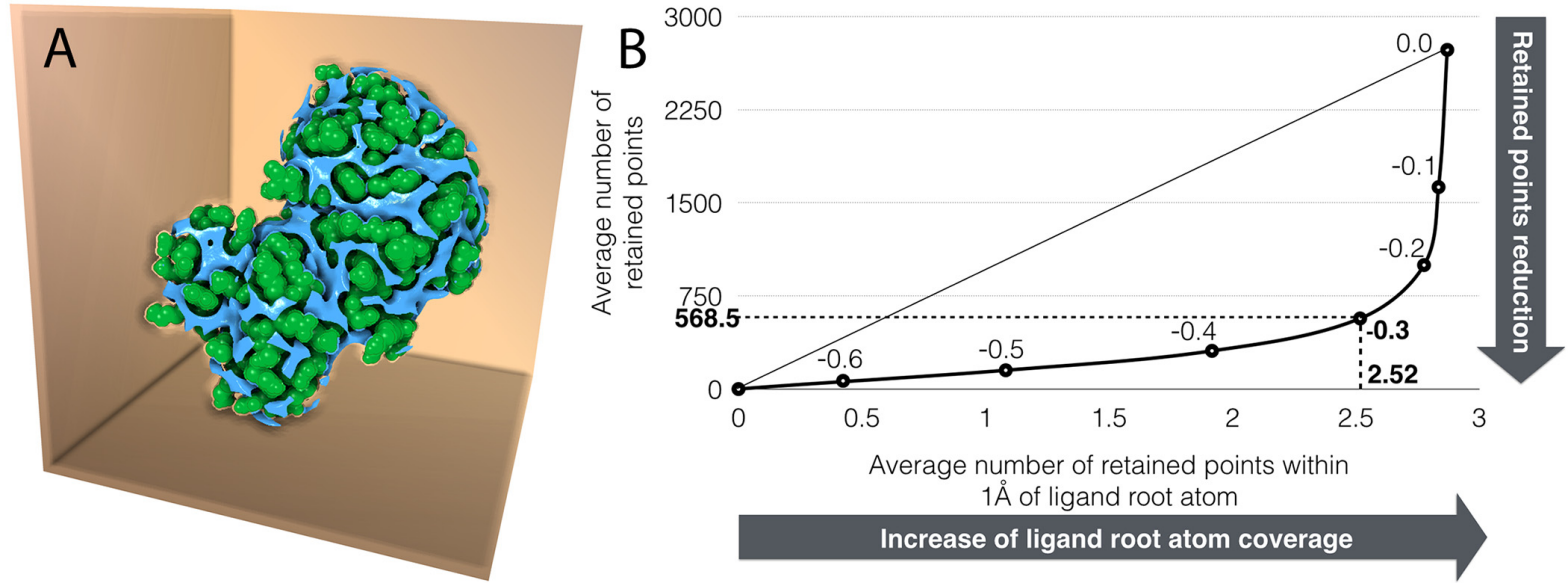
A) Translational points. The surface enclosing points of the carbon affinity map located outside the protein and with carbon affinity less than or equal to -0.3 kcal/mol is shown in blue; protein atomic spheres (with reduced vdW radii) are shown in green. The set of translational points cover grooves and cavities that can accommodate a ligand and provide sensible initial placement points for the ligand root atom. B) Translational points cutoff value selection For energy cutoff values varying from 0.0 to -0.6 kcal/mol in decrements of 0.1 kcal/mol, the average number of grid points retained is plotted against the average number of retained grid points within 1Å of the ligand root atom. The average is computed over the 85 systems in the Astex Diverse set. Lower energy cutoff values produce fewer translational points, however they increase the chance of discarding points surrounding the ligand root atom (i.e. reducing coverage of the root). The value of -0.3 kcal/mol is the closest to the curve’s inflection point and was selected as the best cutoff value to maximize the reduction in retained points and maximize the coverage of the ligand root atom.

In *ADFR*, the ligand is placed into the receptor by translating a central atom of the ligand (called the root atom) to a point inside the docking box. For a given docking solution in the GA this translation is stored in the ligand translation gene. When the initial population is created, this translation gene of individuals in the population is selected from a set of grid points, namely *translational points*. Translational points are defined by analyzing the carbon affinity map and selecting all points with affinity of -0.3 kcal/mol or better located outside the receptor volume (Fig 4A). The optimal value for the affinity cutoff of -0.3 kcal/mol was identified by analyzing grid maps calculated for the 85 complexes from the Astex Diverse Set [33] using a cubic box of size 26.6Å centered on the ligand geometric center. This analysis showed that down-sampling translational points to 1.125Å spacing (i.e., thrice the default grid map spacing) and filtering points with affinity of less than or equal to -0.3 kcal/mol provides the best trade-off between coverage of root atoms in the bound ligands and the reduction of the number of translational points (see Fig 4B). More stringent affinity cutoff values would further reduce the number of points, but reduce coverage. This protocol yielded on average 470 translational points (min: 60, max: 752, sigma: 170) from the initial set of 357,911 total grid points (71^3^), corresponding to an average reduction factor of 99.87%. Interestingly, the number of translational points scales with the fraction of the accessible receptor surface area in the docking box rather than the volume of the box. Therefore, the reduction factor is even more dramatic with larger docking boxes such as the ones used for blind docking experiments, where the actual binding region is unknown and the docking box encompasses the entire protein structure [34–36]. While ligand translation is reduced to a discrete set of points in the initial population, the minimizer modifies the translation values freely allowing off grid translations to be achieved.

#### Soft rotamers

Side-chains of amino acids in a protein exist predominantly in a subset of conformations, referred to as rotamers. *ADFR* leverages this information during the search using a rotamer library compiled by Dunbrack [37]. The rotamer library provides a list of the most frequently observed χ angles and their deviations for each amino acid. During the docking, the soft-rotamer mutation operator modifies receptor side-chain conformations by randomly selecting a rotamer (i.e., a collection of χ angles) and adding random deviations to these angles, based on χ angle standard deviations provided by the library. The local search procedure modifies these angles freely, thus allowing them to potentially explore all values between 0° and 360°. Thus, the rotamer library is used to introduce a biased sampling of the genes representing flexible receptor side-chains rather than pruning the search space.

### Dataset

We performed docking experiments on three different datasets. The Astex Diverse Set is used to validate the implementation in *ADFR* of the *AutoDock4* scoring function, and quantify the increase in performance of *ADFR*’s GA over the one used by *AutoDock*. While the performance of *AutoDock* has been benchmarked [38] using other data sets such as the Astex Clean Set, we chose to the use the Astex Diverse Set in our study, as it is more recent and has been developed specifically to address the shortcomings of the Astex Clean Set [33]. The Astex Diverse Set contains 85 well-curated protein-ligand complexes, has no overlap with the Astex Clean Set, and is best suited for testing docking programs. We define two additional datasets for assessing cross-docking success rate when docking flexible ligands into *apo* conformations of receptors with explicitly specified flexible side-chains, specifically for cases where severe clashes need to be resolved in order to properly dock the ligand.

#### SEQ17 cross-docking set

This dataset was built specifically to test the ability of *ADFR* to modify receptor side-chains’ conformations to enable correct ligand binding in an *apo* receptor. The dataset was obtained from the SEQ dataset [39], which contains *apo*-*holo* pairs for a diverse set of receptors. First, the receptor side-chains interacting with the ligands (i.e. side-chains with at least one pairwise interaction within 4Å) were identified in the *holo* complex. Backbone atoms of these amino acids were then used to superimpose the *apo* structure onto the *holo* structure, yielding an approximate position of the ligand in the *apo* conformation. Next, side-chains from the superimposed *apo* structure clashing with the ligand were identified as the ones for which the distance between a heavy atom of the ligand and a heavy atom of the receptor is within a distance corresponding to half the sum of the van der Waals radii of these two atoms. This selection yielded 35 complexes. We further eliminated 5 complexes in which the clashes involved backbone or Cβ atoms as these clashes cannot be resolved by side-chain conformational changes. We performed re-docking of the ligands into their rigid native complexes for the remaining 30 complexes and selected the final 17 systems for which both *AutoDock Vina* and *ADFR* successfully re-docked the ligand (RMSD < 2Å). This last reduction was performed to eliminate complexes for which re-docking failure is likely due to scoring function limitation in one or the other program. The maximum deviation observed in Cα positions of residues interacting with the ligand between the *holo* and *apo* complexes in this dataset is 2.19Å, except for β-lactoglobulin (*apo*: 1BSQ, *holo*: 1GX9). In this system, Leu87 in the *apo* conformation clashes with the ligand. This residue is located in a loop region that re-arranges upon binding, resulting in a Cα deviation of 8.23Å with respect to the *apo* structure.

#### CDK2 cross-docking set

This cross-docking dataset was built to provide a substantial set of ligands binding to the same receptor. It was built using structures of Cyclin-Dependent Kinase 2 (CDK2) catalytic domain retrieved from the Protein Data Bank (PDB; [40]). CDK2 is a kinase involved in cell cycle regulation, and therefore targeted for cancer therapy. This dataset was designed to test the influence of a variable number of side-chains on a diverse range of interactions between a series of different ligands bound to a single target. A high-resolution *apo* structure (4EK3, resolution of 1.34Å) was selected along with 52 ligand bound *holo* structures in which one or more side-chains interacting with the ligands presented different conformations with respect to the *apo* structure. The 52 *holo* structures were aligned to the *apo* structure by superimposing the backbone atoms, yielding deviations of up to 2Å between the Cα positions of the side-chains interacting with the ligand. A detailed analysis on backbone deviations is reported in the Supporting Information (S1 Table). The full list of PDB IDs used in this study is provided in Supporting Information (S2 Table).

### Docking

Rotatable ligand bonds are obtained by *ADFR* from the PDBQT files used by both *AutoDock* and *AutoDock Vina*. Flexible receptor side-chains are specified in *ADFR* calculations by listing the corresponding amino acids in the input configuration file.

All RMSD values reported in this paper are computed using the Hungarian matching algorithm [41]. The open source Python implementation of the algorithm (http://software.clapper.org/munkres/) was used to find the optimal pairing between atoms of the same type in the two binding poses for which RMSD is being computed. Details of the implementation are described in Supporting Information (S2 Text). Input ligand structures were randomized in their position, orientation and torsions prior to running dockings, using the *AutoDock Vina* randomization function. This prevents possible biases toward the initial conformation in the search algorithm. Flexible side-chains are not randomized in the initial population in order to start from a reasonable initial receptor conformation. This choice does not create a favorable bias in receptor conformation when cross-docking in the *apo* protein conformation. The population size used for *AutoDock* and *ADFR* was based on the following heuristic: 50 + 10 × *L*_*v*_, where *L*_*v*_ is the number of variables pertaining to the ligand in the genome, i.e. 4 (rotation) + 3 (translation) + N_*LRB*_ (number of ligand rotatable bonds). Details on the structure preparation for the 3 datasets are provided in Supporting Information (S3 Text).

#### Astex re-docking

*AutoDock* was run with default parameters, except for: 1) the inclusion of 1–4 interactions, 2) population sizes obtained from the heuristic described above, and 3) a total of 50 GA runs per docking. For each complex, two separate *AutoDock* runs were performed: one with the default number of energy evaluations of 2.5 million (namely *AD*2.5M), and one with a more thorough search with 25 million evaluations (namely *AD*25M). *AutoDock* terminates its GA evolution when it reaches the specified number of evaluations. *ADFR* was run with default parameters, which are 50 GA runs, population sizes obtained from the heuristic described above, and the inclusion of 1–4 interactions. Affinity maps were generated using *AutoGrid*, for cubic grid boxes (26.6Å on a side, i.e. 71-points on a side spaced at 0.375Å) centered on the bound ligand. These maps were used for docking the ligands using *AutoDock*. The maps were processed as described above to obtain the *ADFR* maps and extract translational points.

#### Cross-dockings

Both *ADFR* and *AutoDock Vina* docking calculations were carried out with their default parameters. In the *ADFR* scoring function, the internal energy of the receptor *E*_*REC*_ = *(E*_*FR-FR*_ *+ E*_*FR-RR*_) is down-weighted by a factor of 1/*NFS*, where *NFS* is the number of flexible side-chains. We use docking grid boxes centered on the ligand, and of size 71×71×71 points with the standard 0.375Å resolution in order to encompass all flexible side-chains. The same docking box was used for *ADFR* and *AutoDock Vina*. The translational points were identified in the carbon affinity map and the *ADFR* maps were generated, by processing the *AutoGrid* maps as described above.

#### SEQ17

For cross-docking the SEQ17 ligands into the *apo* receptor, we identified side-chains to be made flexible as the ones with *apo* receptor side-chain heavy atom beyond Cβ within 4.0Å of a ligand heavy atom. The number of selected flexible side-chains varies from 6 to 14, with 11 to 36 rotatable χ angles. The ligands in this set have between 1 and 16 rotatable bonds. The PDB IDs and side-chains selected to be flexible are reported in Supporting Information (S3 Table). *AutoGrid* maps were calculated using the *apo* structure superimposed to the *holo* receptor and a docking box centered on the geometric center of the ligand in the *holo* complex. *AutoDock Vina* was run with default settings.

#### CDK2

The flexible receptor side-chains were selected by tabulating residues interacting with the ligand in the *holo* complex by defining interacting residues as residues with at least one heavy atom beyond Cβ within 4Å of any ligand heavy atom. The resulting interaction pattern varies considerably in the dataset, with ligands contacting anywhere between 4 and 12 side-chains. We selected the following 3 sets of side-chains to be made flexible during the cross-docking: 1) the smallest number of side-chains contacted by a ligand (2R3I), named *FS4*: Ile10, Lys33, Phe82, Leu134; the largest set of interacting side-chains (2FVD), *FS12*: Ile10, Val18, Lys33, Val64, Phe80, Phe82, Gln85, Asp86, Lys89, Asn132, Leu134, Asp145); and finally *FS10*, containing the 10 amino acids most frequently interacting with the ligands: Ile10, Val18, Lys33, Val64, Phe80, Phe82, Asp86, Lys89, Leu134, Asp145. We cross-docked the 52 ligands first into the rigid *apo* structure, then into the *apo* structure with 4, 10 and 12 flexible side-chains. The flexible side-chain groups FS4, FS10 and FS12 contributed respectively: 10, 22, and 27 receptor variables to the genome. The number of rotatable bonds in the ligands varies from 0 to 13. *AutoGrid* maps were calculated using the *apo* structure for a docking box centered at (25.8, 27.6, 27.5). The same grid box definition was used for *AutoDock Vina* setup, and dockings were performed using default search settings (*Vina*8, with exhaustiveness set to 8), followed by runs performing more exhaustive searches with exhaustiveness set to 20 and 200 (named *Vina*20 and *Vina*200, respectively).

## Results

### Astex Diverse Set re-docking

**Fig 5:**
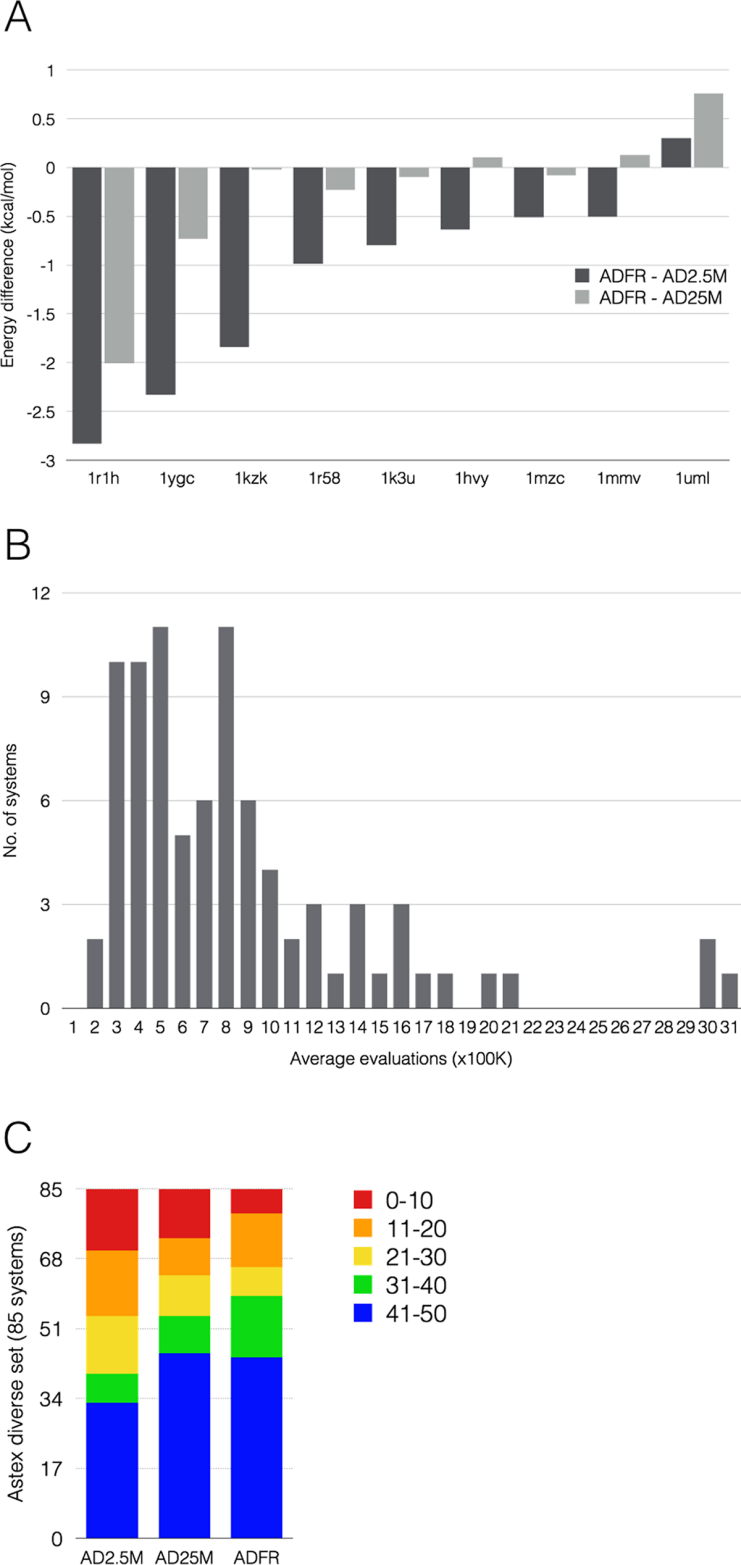
Astex Diverse Set re-docking. A) The bars depict the energy differences between lowest energy solution found by *ADFR* and *AD*2.5M (dark), and *ADFR* and *AD*25M (light). Negative values indicate a lower energy for the *ADFR* solution. Only complexes with at least one of the two differences larger than 0.5 kcal/mol are shown. 1R1H is the only complex where *ADFR* finds a significantly better solution than *AutoDock* (i.e. difference > 2 kcal/mol). B) This histogram shows the distribution of number of evaluations of the scoring function performed by *ADFR* in the GA evolution leading to lowest energy solution. C) Each docking consists of 50 GA evolutions, each producing a solution. The 50 solutions are clustered with an RMSD cutoff of 2Å. In this diagram the 85 complexes are binned based on the cluster size of the lowest energy solution indicating how many of the 50 GA runs identified the pose corresponding to the lowest energy pose found across the 50 runs, i.e. the *reliability* of the GA.

*ADFR* and *AutoDock4* re-dock the ligands of the Astex Diverse Set into their rigid receptors with the following success rates: ADFR: 74%, AD2.5M: 77.65% and AD25M: 73% using an RMSD cutoff of 2Å. We used these docking runs to verify our implementation of the *AutoDock4* scoring function and compare the performances of the search engines of these two programs. Scoring *AutoDock* solutions with *ADFR* and *vice versa* yielded identical results. Moreover, 76 out of 85 solutions (89.4%) have an energetic difference of less than 0.5 kcal/mol between the lowest energy solutions identified by *ADFR* and either AD2.5M or AD25M. For 76 and 80 systems (89.4% and 94.1%) both programs identified the same docking pose (RMSD< 2.0 Å between the ADFR solution and the AD2.5M and AD25M solutions respectively). These results are strong evidence for the fact both programs explore the same energy landscape, thus validating our implementation of the *AutoDock4* scoring function and enabling a direct comparison of the GAs implemented in *AutoDock4* and *ADFR*. We compared the performance of the GA implementations using the following three properties: 1) the best score (i.e. lowest energy) found by the programs indicate the *power* of the search method. 2) The *efficiency* of the search engine pertains to the speed at which it finds the solution. For GA algorithms, this corresponds to the number of evaluations of the objective function (i.e. the scoring function). Finally, 3) since GAs are stochastic algorithms, multiple runs are carried out with different random seed numbers. The number of times a GA run identifies the same best solution, measures the algorithm’s *reliability*. The comparison of solutions used to verify that both programs explore the same energy landscape demonstrates that both search techniques have the same power. The fact that no significant energy improvements have been found by increasing the number of evaluations for *AutoDock* from 2.5 to 25 million confirms that *AutoDock* identified the global minimum after 2.5 million evaluations and both programs reached convergence identifying virtually the same solutions. Fig 5A shows the energy differences for the 9 systems with a difference of more than 0.5 kcal/mol. Only one system has a difference larger than 2.0 kcal/mol. Fig 5B shows that the GA implemented in *ADFR* is more efficient as it identifies the same solutions as *AutoDock*, but only using on average 810 thousand energy evaluations per GA evolution. Only three complexes required more than 2.5 million evaluations. The number of evaluations required by each system shows no correlation with either the number of variables in the genome, or the energy differences between scores obtained by *ADFR* and *AutoDock*. Fig 5C compares the reliability of the 2 GAs. In this figure, the 85 complexes are binned based on the fraction of the 50 runs for which the final pose is within 2.0Å RMSD from the pose with the best score. *AutoDock* shows an increased reliability in runs with 25 million evaluations. However, *ADFR* found the solutions more reliably with 59 complexes found with high reliability (i.e. blue and green bars), versus 54 for *AD*25M and 40 for *AD*2.5M. Conversely, the number of complexes found with low reliability (i.e., red bars) is smaller for *ADFR* (6 complexes) than in *AD*25M (12 complexes) and in *AD*2.5M (15 complexes). Overall, the GA implementation in *ADFR* is more efficient and reliable than the one in *AutoDock*, and its termination criterion is able to limit effectively the number of energy evaluations used during docking, while allocating more evaluations when needed.

### Cross-docking experiments

*AutoDock* has a hard-coded upper limit of 32 rotatable bonds that prevents a direct comparison with *ADFR* on the two datasets used for flexible cross-docking. Moreover, the GA implemented in *AutoDock* is known to lose efficiency for problems with more than ~20 rotatable bonds. *AutoDock Vina* has no implementation limit on the number of rotatable bonds it can search, and it uses the same explicit representation of flexible side-chains as *ADFR*, and finally it is known to have better performance than *AutoDock* for high dimensional searches. Hence it provides a good reference for comparing success rates when docking flexible ligands into receptors with explicitly specified flexible side-chains. For *holo* re-docking, an RMSD value of 2.0Å between experimental and docked structures for ligand atoms is widely accepted for identifying correctly docked poses. When docking a ligand into an *apo* structure, the reference position of the ligand is obtained by superimposing the *holo* and *apo* receptor structures. The alignment is influenced by the differences between the two receptor conformations, and by the subset of atoms used for the superposition. In order to mitigate these approximations, we relax the RMSD cutoff value to 2.5Å in our *apo* cross-docking experiments and analyses. Therefore, a rank of 1 for a solution indicates that the lowest energy solution has an RMSD less than 2.5Å RMSD, while a rank of *N* (*N*>1) indicates that *N*-1 false positive solutions were reported.

#### SEQ17 dataset

Table 1 presents a summary of the SEQ17 cross-docking results. For rigid cross-docking *AutoDock Vina* reports a correct solution for one system: (1IKG) and *ADFR* for two systems: (1IT8 and 1Z6P). The binding pocket in the *apo* conformation of 1IT8 allows for a translation of its small rigid ligand (RMSD 1.26Å), which is sufficient to resolve the clash between the pyrimidine moiety in the ligand and the Phe229 side-chain. The nitrobenzoyl and the phthalic acid moieties of the ligand from 1Z6P have a severe clash with an amino group of Arg193 and Arg310, respectively. These clashes can be resolved in the binding pocket in the *apo* conformation of the receptor by rotating these groups generating a ligand RMSD of 1.86Å. The ranking improved from 9 to 2 when docking with flexible receptor side-chains. The ligand from 1IKG correctly docked by *AutoDock Vina*, severely clashes with the hydroxyl oxygen of Ser62 and is in very close proximity to Thr301 backbone carbonyl oxygen. *AutoDock Vina* finds a solution (rank 3, RMSD of 2.27Å) for the ligand by rotating and translating the peptide bond in the ligand to avoid the clash and makes it a rank 1 solution with RMSD 2.45Å when side-chains are made flexible. A clear improvement in success rate is observed with both programs when side-chains interacting with the ligand in the *apo* conformation are made flexible. *AutoDock Vina* finds 4 top ranking solutions (23.5%) and *ADFR* finds 5 (29.4%). *AutoDock Vina* reports an additional 2 solutions ranking 4 and 6 for a total of 6 receptors for which it sees solutions (35.3%). *ADFR* reports 4 more systems with ranks 2 and 3 (52.9%) and another 3 systems with solutions ranking 14 (70.6%). Table 1 also provides the difference in reported scores between the best correct solution and the best incorrect solution (ΔScore) for both *ADFR* and *AutoDock Vina*. These differences provide a level of confidence for the solution’s rank. A small ΔScore indicates the existence of an alternate pose that is very close energetically. If this difference is below the scoring function’s intrinsic error the two solutions are indistinguishable, and their relative ranking is not informative. A large ΔScore on the other hand indicates that the rank is more informative.

Considering the top 10 ranking solutions *ADFR* is successful for 52.9% of the systems and *AutoDock Vina* for 35.3%. The RMSD values of the best scoring ligand pose and the RMSD of the best scoring solution and lowest energy correctly docked solution (if different) are provided in the Supporting Information (S4 Table).

**Table 1:** SEQ17 cross-docking into *apo* conformations with receptor side-chains.

					Rank (RMSD < = 2.5 A)					
SEQ17		Number of			Rigid Cross Docking		Flexible Cross Docking			
		Ligand	Receptor							
*holo*	*apo*	Rot. Bonds	Flexible side-chains	Rot. χ angles	Vina	ADFR	Vina	ADFR	ΔScore (Vina)	ΔScore (ADFR)
1IT8	1IQ8	1	6	11		1	1	1	0.1	0.711
1K4H	1PUD	5	9	19			1	1	0.0	1.675
1GX9	1BSQ	5	11	27				1		1.916
2H8H	1FMK	4	8	16				1		0.054
3JRX	2HJW	6	12	30			1	1	0.3	0.607
1Z6P	2GPN	7	8	24		9		2		0.318
1AQ1	1HCL	2	9	22				2		1.512
1QKJ	2BGT	8	9	24				3		0.763
1LNM	1KXO	3	12	27			4	3	0.2	0.644
1IKG	3PTE	13	14	28	3		1	14	0.1	2.314
1C1H	1DOZ	8	12	31				14		3.342
3ERK	1ERK	4	8	20				14		2.114
1RBP	1BRQ	6	8	18			6		1.9	
1BR5	1RTC	7	7	16						
1YXT	1XQZ	6	11	27						
1ZG3	1ZHF	4	8	18						
2A9K	2A78	16	13	36						

The table lists the PDB IDs for the 17 *apo* and *holo* structure pairs in the dataset, the number of rotatable bonds in the ligand, the number of flexible receptor side-chains and the corresponding number of rotatable χ angles. Ranks for solutions with RMSD less than 2.5Å are reported. Empty cells denote no solution found within this RMSD cutoff.

#### CDK2

We cross-docked the 52 ligands from the CDK2 dataset into the *apo* structure with various levels of receptor flexibility, ranging from 0 to 12 flexible side-chains.

The cross-docking results are summarized in Table 2. The table shows the percentage of complexes for which the top ranking solution is correct (rank 1) as well as the percentage of systems for which a correct solution is in the top ten ranking solutions. *ADFR* outperforms *AutoDock Vina* in all the tests. In rigid cross-docking, the relatively small number of degrees of freedom (between 7 and 20) is unlikely to be the reason for the lower performance of *AutoDock Vina*. Hence, the better *ADFR* results suggest that the *AutoDock* scoring function might be less sensitive to small perturbations in the shape of the pocket, compared to the one implemented in *AutoDock Vina*. Overall docking performance improved for both programs with the increase of receptor flexibility in the binding site. Results with all flexible side-chain groups (FS4, FS10 and FS12) show that *ADFR* consistently achieves a better success rate using both “best ranked” and “top 10 results” metrics. RMSD values for the best scoring ligand pose and for the best scoring correctly docked pose (if different) are provided in the Supporting Information (S5 Table). Increasing *AutoDock Vina* exhaustiveness showed no improvement in success rate, with even worse results in some cases (Supporting Information–S6 Table).

**Table 2:** Cross-docking results comparison between *ADFR* and *AutoDock Vina* with 0, 4, 10, 12 flexible receptor side-chains.

Flexible Side-Chains		Solutions (RMSD < = 2.5Å)			
		Rank 1		Rank < 10	
# Active	# Rot. χ angles	Vina8	ADFR	Vina8	ADFR
0	0	4 (7.7%)	12 (23.1%)	7 (13.5%)	24 (46.2%)
4	10	12 (23.1%)	19 (36.5%)	26 (50.0%)	40 (76.9%)
10	22	14 (26.9%)	21 (40.4%)	30 (57.7%)	41 (78.8%)
12	27	16 (30.8%)	23 (44.2%)	32 (61.5%)	40 (76.9%)

The left section (“Flexible side-chains”) show the number of side-chains considered flexible in each docking and the corresponding number of rotatable χ angles. The right section reports the number of systems (and percentage) for which the ligand-RMSD of the lowest energy solutions found by *ADFR* and *AutoDock Vina* are less than 2.5Å (rank 1) or a correctly docked solution is within the 10 top solutions (rank < 10).

#### Efficiency and complexity considerations

**Fig 6:**
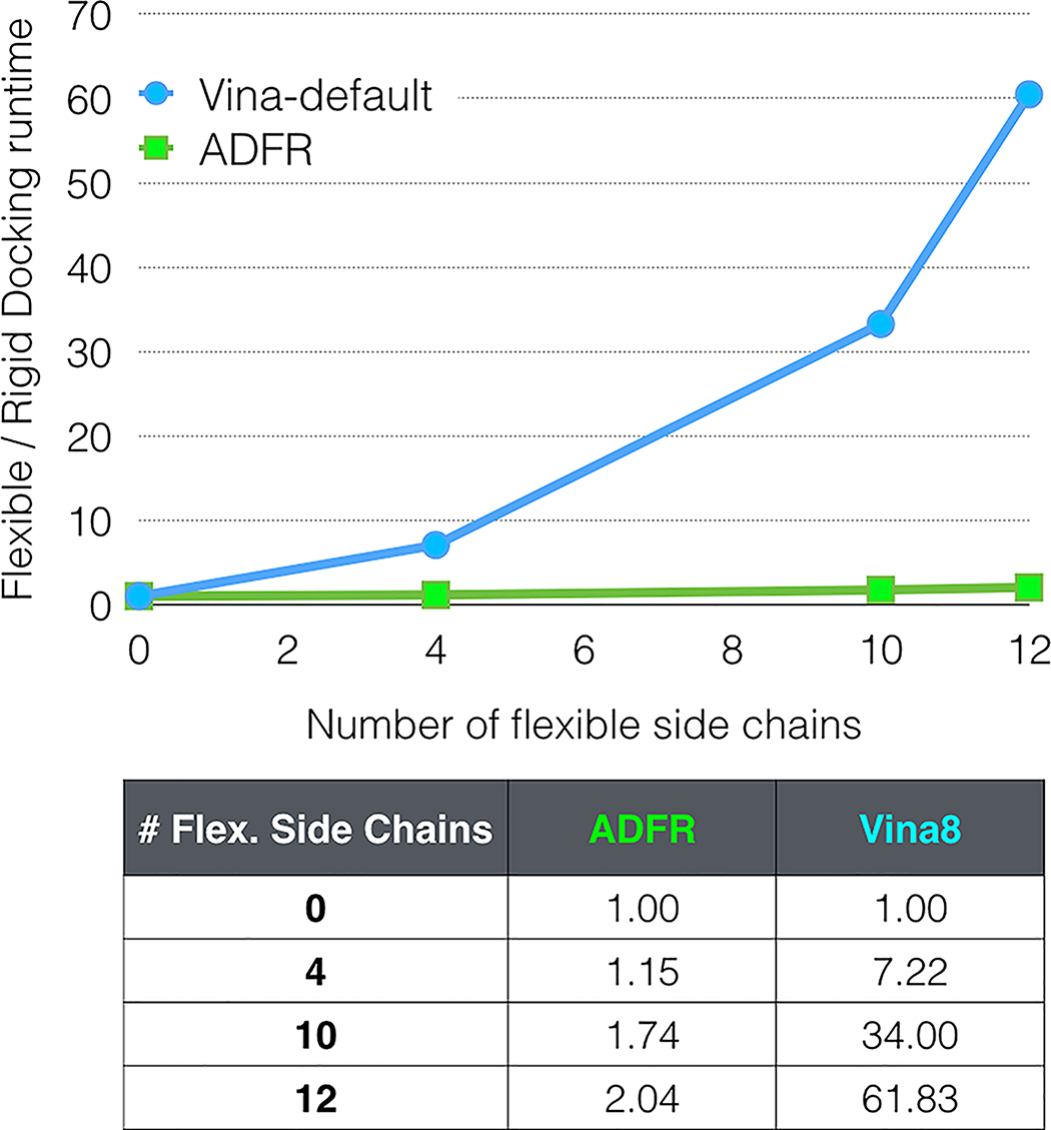
Scaling of docking runtimes as function of the number of flexible receptor side-chains. The Y-axis represents multiples of the rigid cross-docking runtimes. The times used in this graph are averages taken over all docking runs for the 52 complexes of the CDK2 cross-docking experiments. For *AutoDock Vina* the times corresponding to the default exhaustiveness 8 are used. The X-axis indicates the number of flexible receptor side-chains. *ADFR* scales by a factor of 2, while *Vina*8 scales by a factor of 62, when 12 protein side-chains are made flexible.

Unlike *AutoDock*, *AutoDock Vina* uses a different scoring function than *ADFR*. In addition, it does not report the number of evaluations of this scoring function, thus preventing a direct comparison in terms of efficiency. The average wall time for docking with 12 flexible receptor side-chains on a single core on XEON-EMT processor are: 1.85 hours for *AutoDock Vina* with exhaustiveness 8, and an average of 8.5 hours per GA evolution for the Python implementation of *ADFR*. The run-time for *AutoDock Vina* scales exponentially as the number of flexible receptor side-chains taking an average of 1.8, 13.0, 61.2 and 111.3 minutes for RCD, FCD4, FCD10 and FCD12 respectively. On the other hand, the run-time for *ADFR* increases linearly from 4.2 hours on average for RCD to 4.8, 7.3 and 8.6 hours per GA evolution for FCD4, FCD10 and FCD12 respectively. Fig 6 shows the ratios of flexible docking run-times (FS4, FS10 and FS12) to rigid docking run-times for both programs using average run-times computed over the 52 systems. Docking into a receptor with 12 flexible side-chains takes more than 60 times longer than docking into a rigid receptor using *AutoDock Vina* with default search settings (*Vina*8). Higher exhaustiveness settings *Vina*20 and *Vina*200 increased run-times by 2-fold, and more than 20-fold than default *Vina*8, respectively (Supporting Information–S7 Table). On the other hand, the *ADFR* run-time with 12 flexible side-chains is only twice the run-time for rigid docking.

#### Receptor side-chain motions

**Fig 7:**
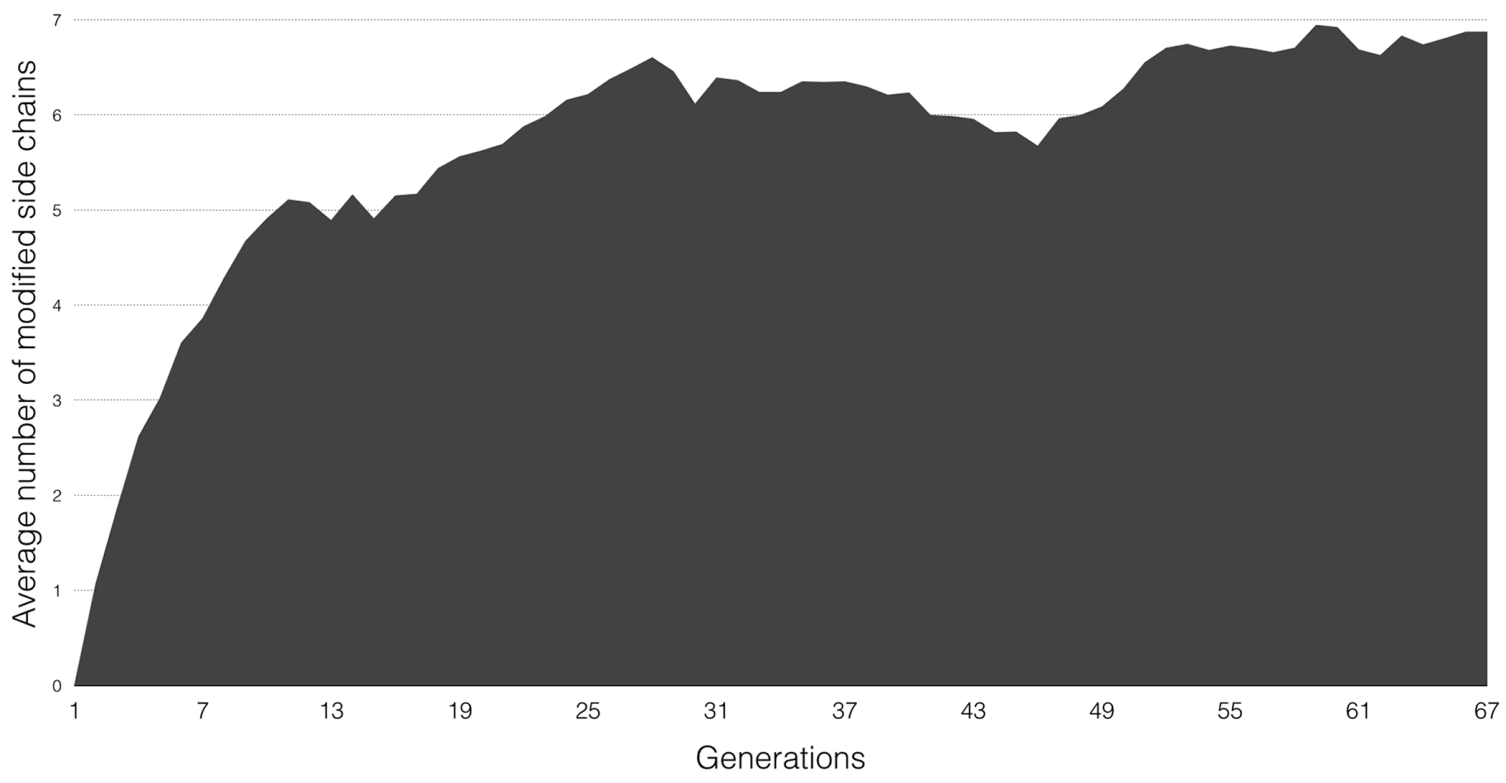
Frequency of receptor side-chain changes in the GA population during a successful docking of the 4EK6 ligand docked into the corresponding 4EK3 *apo* receptor with 12 flexible side-chains. The figure plots the evolution of the average number of receptor side-chains with a modified conformation over successive generations of the GA optimization. In the initial population all receptor side-chains are in the *apo* conformation. The number of side-chains changing rotameric state in individuals of the optimized population quickly increases in the first few generations and reaches a plateau. This profile is typical and observed in all runs for all system.

Each flexible receptor side-chain starts the docking in the conformation of the input structure (i.e. the *apo* conformation in our cross-dockings). This conformation is modified during the docking procedure by the mutation operator and the local search procedure. The mutation assigns new conformations randomly using χ angles and deviations from the rotamer library. The local search procedure on the other hand freely modifies these χ angles. Subsets of flexible receptor side-chains can also be exchanged between individuals through a crossover operation. We analyzed all populations optimized by the GA during the FS12 docking runs to gain insight into the number of side-chains changing rotameric state over the course of the evolution. A side-chain conformation is deemed changed if at least one of its χ angles deviates by at least 50° from the input structure. This value corresponds to the smallest difference in χ angles in the rotamer library used by *ADFR*. The analysis of the data across all generations in all 50 runs for all 52 complexes reveals an interesting emergent property of the GA. While individuals with up to 12 modified side-chains are seen but are very rare, the average number of modified side-chains per individual is 5.6, out of 12. Fig 7 shows a typical profile of the number of modified side-chains over consecutive generations of the GA. The figure shows that this number rises rapidly from 0 (in the initial population) to reach a plateau. This behavior is in agreement with the analysis of Gaudreault and co-workers [39], which reported that only 5 or fewer side-chains alter their rotameric conformation within an angular cutoff of 60° upon ligand binding.

#### Flexible receptor side-chain interactions with the ligand

**Fig 8:**
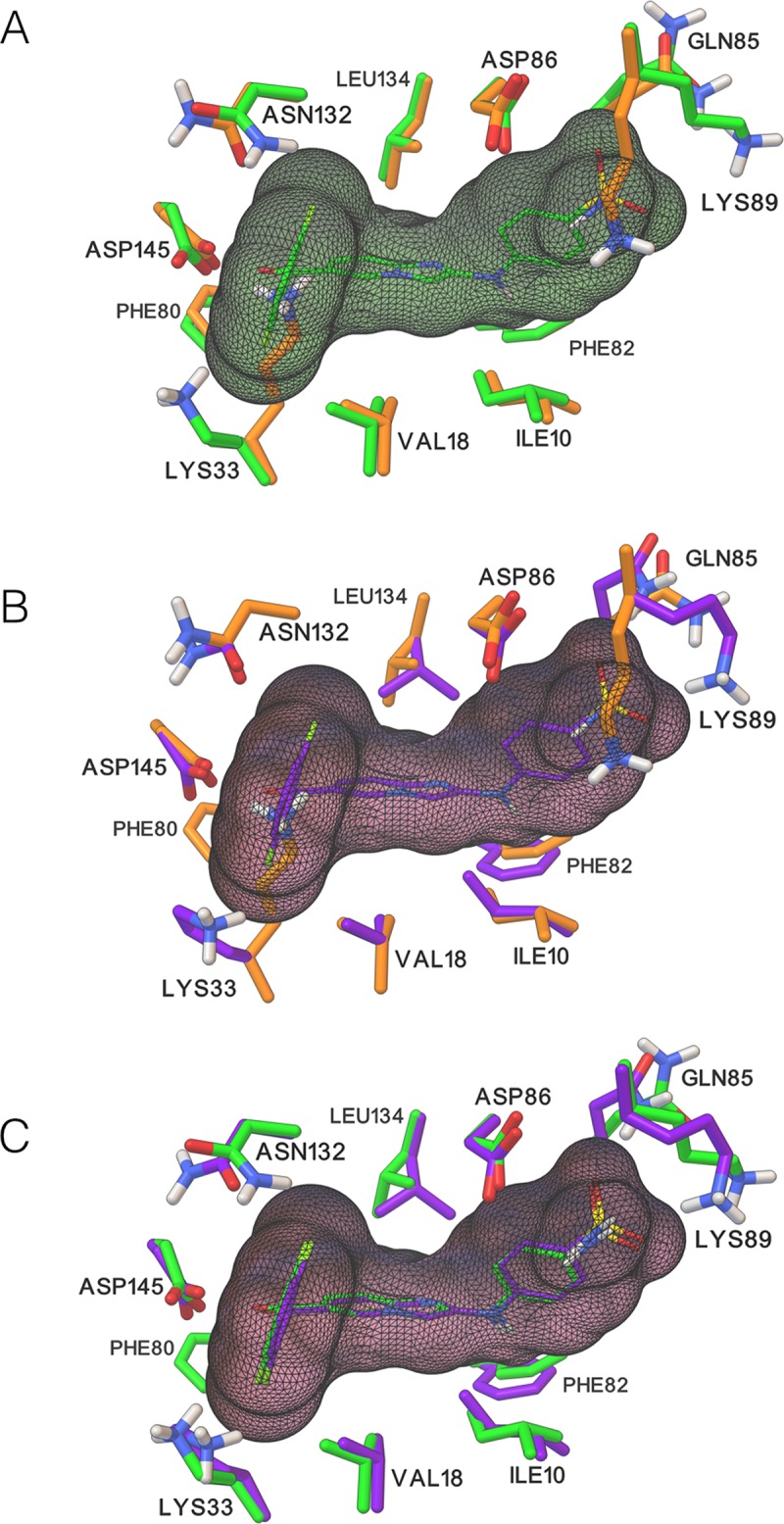
Comparison of side-chain conformations between *apo*, *holo*, and successfully docked solution. This figure provides a pairwise comparison of the conformations of the *apo* (4EK3), *holo* complex (1YKR), and the 1YKR ligand docked solution with the 12 flexible receptor side-chains displayed as ball-and-sticks. A) *Apo* vs. *holo*: The native bound ligand is displayed as sticks with green carbon atoms along with a partially transparent green molecular surface. The 2 lysine side-chains in the *apo* conformation severely overlap with the space occupied by the ligand. B) Docked vs. *apo*. The docked solution is shown with purple carbon atoms and partially transparent ligand molecular surface. The *apo* structure is shown with orange carbon atoms. All 12 side-chains in the docked solution adopt conformations different from the initial *apo* conformation. Most of them settle for conformations corresponding to small adjustments while others adopt substantially different conformations to resolve steric clashes (Lys33 and Lys89). C) The docked solution (purple carbon atoms) is shown with the *holo* receptor (green carbon atoms). The ligand is docked perfectly (RMSD from the crystallographic structure is 0.34Å) and the receptor side-chains changed their conformations to accommodate the ligand binding in the correct binding mode.

While the measure of success for our cross-docking experiment is the ligand atoms RMSD with respect to the approximate ligand superimposed to the *apo* structure, it is also important to understand how flexible receptor side-chains change conformation upon binding the ligand. Fig 8 provides an example of conformational changes in the receptor between the *apo* structure (4EK3), the *holo* complex (1YKR), and the best docked solution (*apo* receptor with 12 flexible side-chains). Fig 8A shows that there are no significant deviations in Cα positions between the *apo* and *holo* structures. Lys33 and Lys89 in their *apo* conformation have a severe clash with the ligand in its *holo* conformation, thereby preventing its successful cross-docking into the rigid *apo* receptor (RMSD value of 5.96Å for the best energy solution). Also, Asp132 and Glu85 are flipped, while the remainder of the side-chains retain their *apo* conformation upon ligand binding. Fig 8B shows the docked ligand in the *apo* structure. All flexible side-chains have adjusted their positions. In particular, the two lysines have moved to a conformation closer to their *holo* conformation to enable the ligand to bind with an RMSD of 0.34Å. Fig 8C shows the docked solution with the *holo* complex. The two lysines have moved closer to their *holo* conformation. The difference in conformation of Gln85 could be due to the fact that in X-ray structures the similarity in electron density of oxygen and nitrogen limits the ability to assign the correct side-chain orientation for the amido groups of Asn and Gln. In the docked solution, Gln85 flips its amido group between the *holo* and *apo* structure because of its interaction (or lack thereof) with Lys89. Moreover, the amine group of Gln85 forms a hydrogen bond with a water molecule in the *holo* structure. In the docked solution this side-chains remains close to its *apo* conformation despite Lys89 moving closer to its *holo* conformation and interacts with the ligand sulphonate group to make a hydrogen bond. Hence, the conformation adopted by Gln85 is likely due to down-weighting the receptor energy term and the lack of explicit water molecules during the docking. A more detailed analysis of the side-chain interactions with the ligand across the set of successfully cross-docked solutions is described below.

**Fig 9:**
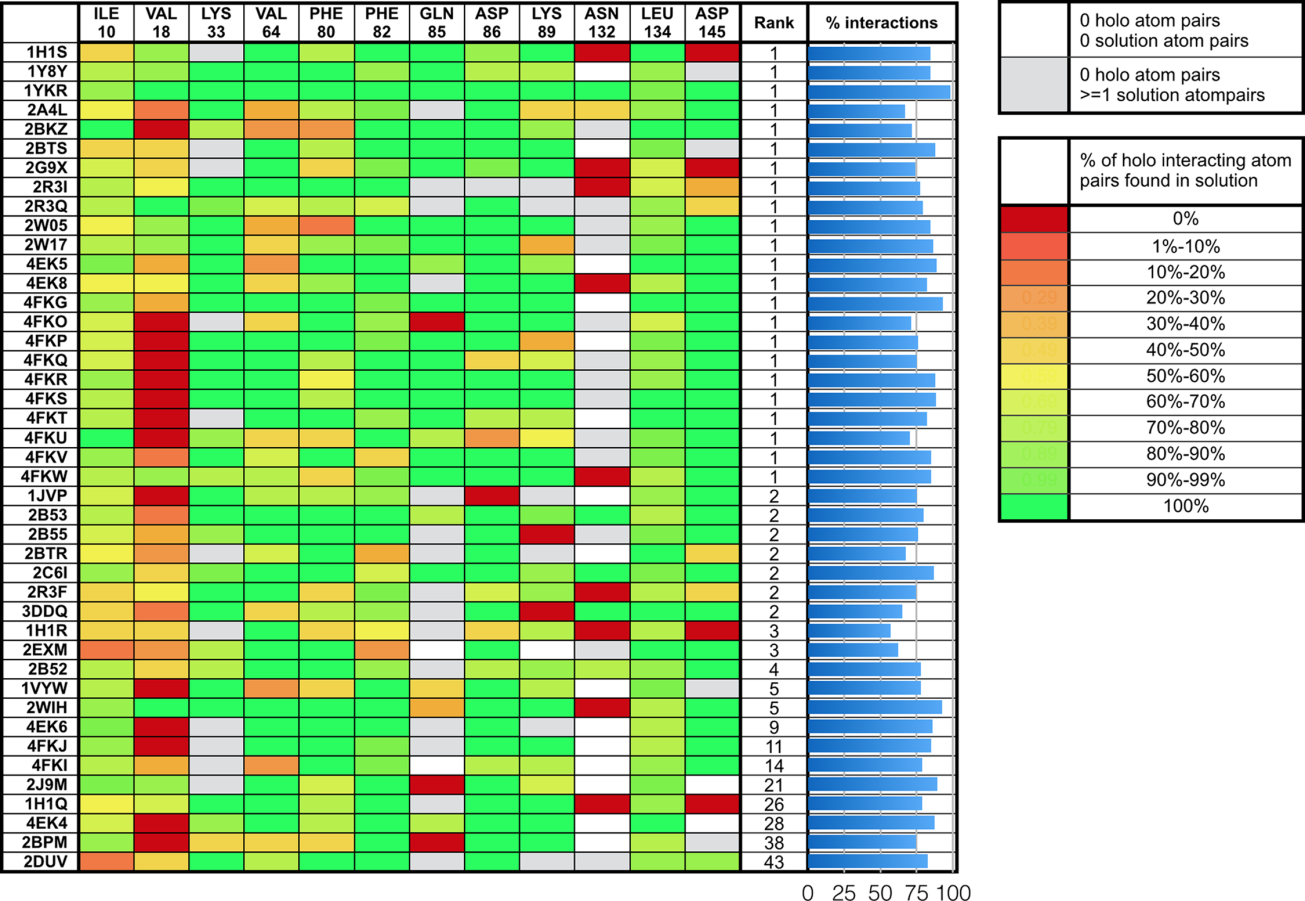
Heat map of ligand-flexible receptor atomic contacts reproduced in docked poses. The 43 systems reported in this table are the ones for which ADFR correctly reports the docked solution (i.e. ligand RMSD < 2.5Å). The rank of the solution among 50 GA runs is reported. White cells correspond to flexible side-chains not interacting with the ligand in either the *holo* or the docked complex. Grey cells indicate interactions formed in the docked solution, which do not exist in the *holo* complex. The remainder of the cells is colored using a red to green color scale indicating the percentage of *holo* interacting atomic pairs reproduced by the docked solution. A green cell (rate of 100%) indicates that every pairwise atomic interaction between ligand atoms and the side-chain atoms of the residue corresponding to that cell are reproduced in the docked solution. The histogram displays the percentage of *holo* interactions that are reproduced across all 12 side-chains for every ligand. The ligand reproduced at least 57.1% of all the interacting pairs in the *holo* complex, with an average of 79.8% interactions.

We considered different metrics in order to assess the success in modeling the induced fit when docking in the *apo* structure with 12 flexible side-chains (FS12 calculation). We found that the RMSD of moving receptor atoms does not provide a good measure of the nature of the receptor side-chain motions (see Discussion section). Instead, we tabulate atomic pairwise interactions in the *holo* complex and analyze the recovery rate of these interactions in the 43 systems for which *ADFR* successfully generated at least one correct solution (RMSD < 2.5Å). We define a pair of ligand-moving receptor atoms as interacting if they are located within 5Å of each other. Symmetry in ligand and receptor atoms was considered when matching pairwise atomic interactions. Fig 9 shows the percentage of *holo* pairwise atomic interactions reproduced in docked solutions for the 43 complexes used for this analysis. A rate of 100% (green) in a cell of this heat map indicates that every pairwise atomic interaction between ligand and the side-chain atoms is reproduced in the docked solution. On average, every docked pose reproduces 79.8% of pairwise *holo* interactions, with a minimum of 57.1% interactions (for 3DDQ). For each pose, its rank in the 50 solutions prior to clustering is shown.

Some failures in reproducing the *holo* interaction patterns can be attributed to backbone atom deviations between *apo* and *holo*. The highest deviation values were found for Ile10, Val18 and Lys33 (about 2Å). The side-chains of Lys33 and Ile10 have enough flexibility to accommodate such variations. Val18, on the other hand, has only one χ angle, which rotates 2 carbon atoms in a plane parallel to the plane containing the ligand atoms. Thus side-chain flexibility on Val18 cannot compensate for the backbone shift away from the ligand observed in the *apo* structure.

For 45.1% of the moving side-chain (across the 43 complexes), all interactions observed in the *holo* complex are reproduced in the docked pose, and 89.7% side-chains reproduce at least half of their *holo* interactions. 11.2% of the receptor side-chains that are not contacting the ligand in the *holo* structure created at least one interaction with the ligand (grey cells). Most of these additional interactions in the docked solutions are accounted for by Lys33 (14), Gln85 (16), and Asn132 (32). For these side-chains, the benefit of a weak interaction with the ligand outweighs the downscaled receptor-receptor interactions these side-chains make in the *apo* conformation.

### Impact of down-weighting the receptor internal energy

**Fig 10:**
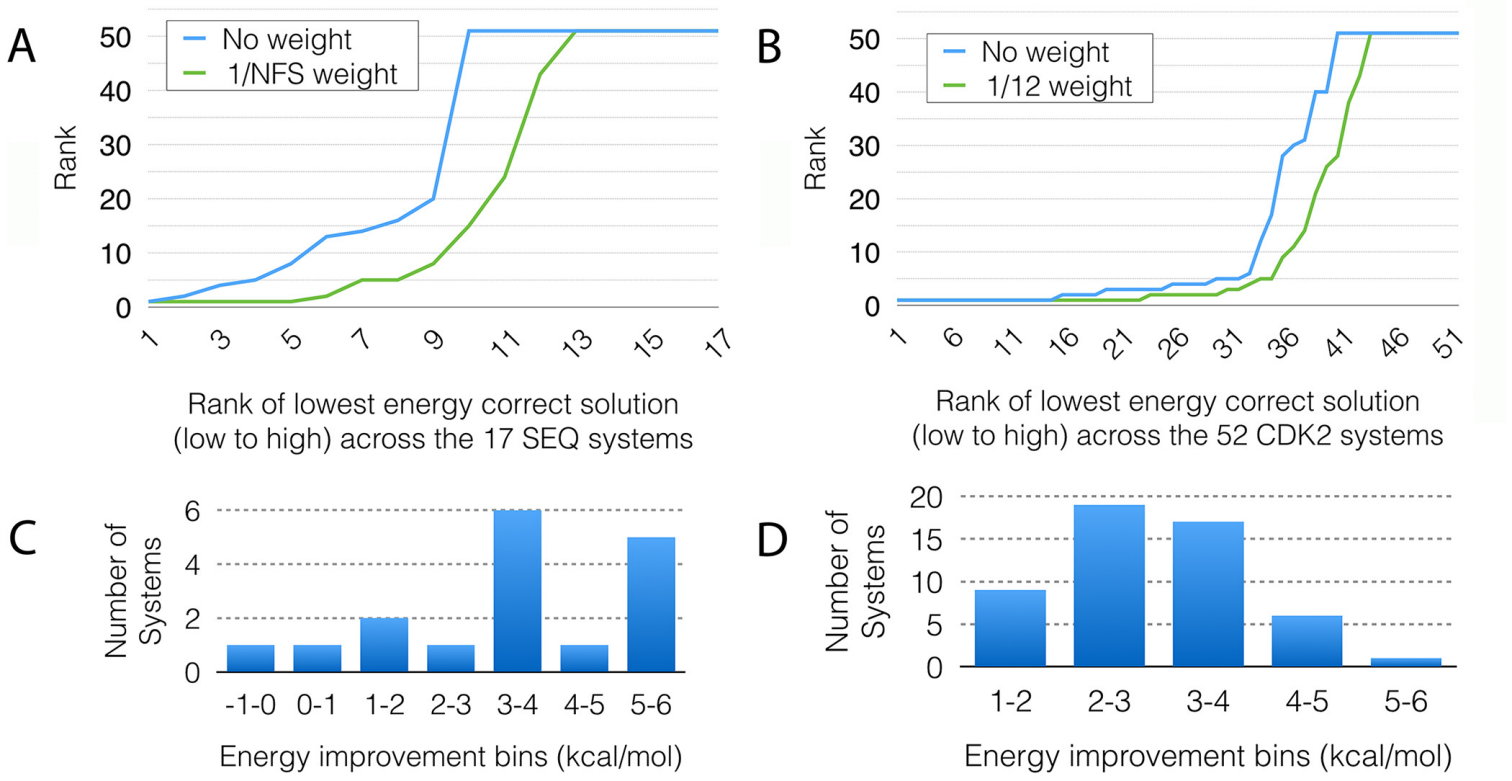
Impact of down-weighting the receptor internal energy. A) and B) Sorted ranks of the correct docked solutions without scaling the receptor energy (blue) and with a scaling factor of 1/*NFS* (green) where *NFS* is the number of flexible receptor side-chains, for the SEQ17 and CDK2 FS12 cross-docking calculations respectively. Overall down-weighting the receptor energy improves the rank of the lowest-energy correct solution. The top horizontal line (Rank 51) in the plots represents data points that did not find the solution in the 50 docking runs. C) and D) Distributions of improvements in receptor-ligand interaction energies (*E*_*R-L*_) in kcal/mol, when the internal energy of the receptor is down-weighted in the scoring functions for SEQ17 and the CDK2 FS12 calculation respectively.

When the moving receptor atoms greatly outnumber the ligand atoms, the receptor internal energy component (*E*_*REC*_ = *E*_*FR-FR*_ *+ E*_*FR-RR*_) dominates the score. Without correction, this leads the GA to primarily optimize the conformation of the flexible receptor rather than the ligand-receptor interactions (*E*_*LIG*_ = *E*_*L-FR*_ *+ E*_*L-RR*_). Our results show that down weighting the receptor contribution *E*_*REC*_ by the inverse of the number of flexible side-chains (e.g., *E*_*REC*_/12, for the FS12 set) results in an overall improvement in ranks of correct solutions. Fig 10A and 10B show the sorted ranks of the first correct solution without (blue) and with (green) weighted *E*_*REC*_ values for the SEQ17 and CDK2 FS12 cross-dockings respectively. We observe an overall improvement in ranks and, with an increased success rate from 61.5% to 76.9% in the CDK2 dataset and 35.2% to 52.9% in SEQ17 when considering the top 10 solutions. At the same time, the interaction energy improved significantly in docked solutions obtained by down-weighting the receptor internal energy. The improvement ranges from 1 to 7 kcal/mol (Fig 10C and 10D) with most complexes gaining 3 to 4 kcal/mol. This result supports the idea that without attenuating the receptor internal energy component, the GA fails to optimize the ligand-receptor interactions increasing the rate of false positives. It also indicates that applying separate weights for the different energy terms (i.e. *E*_*REC*_ vs. *E*_*LIG*_ vs. *E*_*REC-LIG*_) allow shifting the focus of the search engine. In preliminary results on docking very large ligands such as peptides, we observe a reversed situation with the ligand internal energy dominating the score. Hence more sophisticated approaches for balancing energetic contributions of the various atom sets are needed. We are currently working on a more elaborate scheme for normalizing the various terms of the scoring function and deriving appropriate weight to apply to these normalized terms of the scoring function.

## Discussion

In this paper we introduce a new docking software, *AutoDockFR* (or *ADFR*), for docking flexible ligands into receptors with explicitly specified flexibility. We show that *ADFR* outperforms the widely used docking programs *AutoDock* and *AutoDock Vina* in various docking experiments and introduce two sets of protein complexes that require substantial conformational changes in receptor side-chains for successfully docking the ligand into the *apo* conformation.

While AutoDock could handle flexible receptor side-chains explicitly since version 3.05 [42], its GA is known to perform best when less than 20 bonds are made rotatable, effectively limiting this option to 1 to 2 flexible side-chains. The re-docking of flexible ligands into the Astex Diverse Set provides quantitative assessment of differences between the new GA implemented in *ADFR* and the one implemented in *AutoDock*, showing increased efficiency and reliability. This performance increase is obtained through a combination of techniques including: population clustering, efficient termination criteria, and the encoding of knowledge such as translational points and soft rotamers helping the GA to identify good solutions faster. Other docking software (e.g. Gold, Fitted) use techniques similar to translational points and rotamers for pruning the search space. However, in *ADFR* this information is used to sample promising areas of the search space more frequently while retaining continuous sampling of the entire search space, rather than pruning it. We performed cross-docking experiments on two datasets. The SEQ17 dataset focuses on receptor diversity, while CDK2 focuses on ligand diversity. Both these cases are relevant docking scenarios and the choice of an *apo* conformation as the target provides a realistic scenario of the challenges associated with induced fit simulation.

The SEQ17 dataset consists of 17 receptors in which substantial side-chain motion is necessary in the *apo* conformation for docking the ligand. The largely failed cross-docking observed with rigid *apo* conformation of the receptor confirms that the SEQ17 provides a challenging set of complexes. The cross-docking experiment demonstrates that adding flexibility to the receptor increases docking success rate. *ADFR* reports solutions for 70.6% for the complexes and solutions with rank less than 10 for 52.3% of these complexes, outperforming *AutoDock Vina* success rate of 35.3%. Refinements to the scoring function could further increase the docking success rate of ADFR in the future. In particular, the addition of rotatable terminal hydrogen atoms in flexible receptor side-chains will improve docking accuracy on this dataset as a substantial number of moving receptor side-chains contain such hydrogen atoms.

The cross-docking of the CDK2 dataset shows a higher success rate for *ADFR* and a substantial improvement over *AutoDock Vina*. This docking scenario is different from the SEQ17 dataset as we are using a set of 52 ligands docked into a single *apo* conformation of the receptor. We show that *ADFR* outperforms *AutoDock Vina* in all scenarios of flexible docking, when we cross-dock 52 CDK2 ligands in a CDK2 *apo* receptor conformation with 0, 4, 10, and 12 flexible side-chains. We performed a detailed analysis receptor side-chain motions and the impact of adding receptor flexibility on docking success rate.

### Receptor side-chain motion analysis

The RMSD of moving receptor atoms is not a suitable metric for gaining insight into receptor side-chain motions for the following reasons. First, in many cases, including the SEQ17 and CDK2 datasets used here, only a small subset of receptor side-chains interacting with the ligand undergo a substantial change in their conformation. The contributions of these side-chains to the RMSD of moving receptor atoms is outweighed by the contributions of a larger number of side-chain staying close to their initial conformation. Second, computing RMSD requires a reference conformation, which is the target conformation to be achieved for success. Ideally the *holo* conformation should be induced when docking a ligand into an *apo* conformation. However, this would require the receptor to be fully flexible. In our experiments only side-chains interacting with the ligand can move. These side-chains exist in the context of an *apo* conformation; hence they are not always expected to achieve the *holo* conformation. For example, side-chains not interacting with a particular ligand have no reason to deviate much from their *apo* conformation. Moreover, even small backbone perturbations can change the Cα-Cβ vector potentially forcing a side-chain to adopt an alternate conformation to interact with the ligand. For these reasons, we used pairwise atomic interactions between ligand and moving receptor atoms in the *holo* complex to assess receptor side-chains motions. Results show that an average of 79.8% of these atomic pairwise interactions are recovered in docked solutions, showing that the flexible receptor side-chains move to re-create the interaction pattern observed in the *holo* complex.

### Impact of adding receptor flexibility on docking success rate

**Fig 11:**
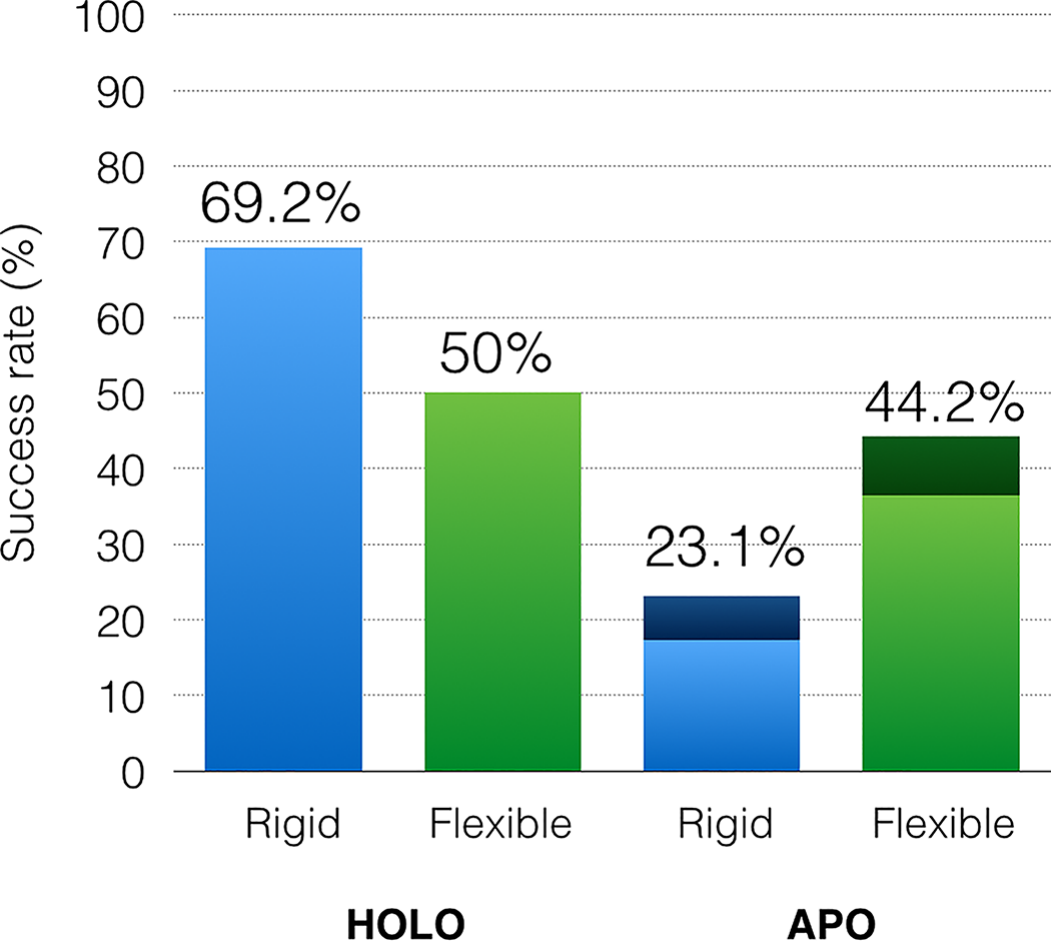
Impact of making 12 receptor side-chains flexible when docking ligands into the native *holo* receptor and the *apo* receptor. An expected loss of accuracy is observed when making the native *holo* receptor flexible, reflecting shortcomings in the scoring function and search method. Adding flexibility to the *apo* receptor, however, improves the docking success rate. *Holo* docking success rates are shown for ligand RMSD < 2Å. The success rate for *apo* cross-docking increases from 17.3% to 36.5% with a 2.0 Å RMSD cutoff. This success rate increases from 23.1% to 44.2% when using a 2.5Å RMSD cutoff (darker shade bars).

Fig 11 shows the success rates (i.e. percentages of CDK2 complexes for which the top ranking solutions have an RMSD from the crystallographic structure of less than 2Å (*holo*) and 2.5Å (*apo*)) achieved by *ADFR* when docking into both the *apo* and *holo* structures, rigidly and with 12 flexible side-chains. An expected decrease in performance is observed between docking a ligand into its rigid native *holo* receptor (69.2%) versus docking it into the same receptor with flexible side-chains (50%). This can be attributed to shortcomings of the model (e.g., implicit solvent, scoring function limitations) that result in false positives out-scoring the correct solution. However, adding flexible side-chains improves the results considerably when cross-docking ligands into the *apo* structure, increasing the success rate from 23% to 44%.

Cross-docking into the *apo* structure is a more challenging task compared to docking a ligand into a native or non-native *holo* structure [43], and represents a realistic scenario where candidate molecules are evaluated for their capability to bind into a given structure. Both cross-docking tests were performed using *apo* conformations. Further, the ability to handle as many as 12 side-chains reduces the burden of having to choose which side-chains should be considered flexible before running the docking calculation. The SEQ17 and CDK2 datasets are representative of specific, but relevant type of receptor conformational change. They have shown to be challenging for *AutoDock Vina* and *ADFR*. Comparing the merits of the various other approaches described in the introduction for dealing with receptor flexibility on these datasets will be interesting but is beyond the scope of this paper.

#### Open architecture for methods development

The open architecture of *ADFR* is designed to incorporate a variety of motion objects and we are working on adding motion operators for local and global receptor backbone motion. This architecture supports exploring new techniques, but the python implementation gives it poor performances in execution time. The current implementation of *ADFR* is on average 230 times slower than the highly optimized C++ code of *AutoDock Vina* when docking into a receptor with 12 flexible side-chains. Currently, a GA evolution requires an average of 8.5 hours for receptors with 12 flexible side-chains. Hence, at this point, *ADFR* is only suitable for users having access to substantial computational resources (i.e. large clusters) in order to perform independent GA evolutions in parallel on different processors. We are working on a C++ implementation that will dramatically reduce execution times.

In summary, we demonstrate that adding flexibility to the *apo* conformation of receptors increases docking success rate and that *ADFR* outperforms *AutoDock Vina* on receptors with up to 12 flexible side-chains. For docking approaches explicitly specifying receptor flexibility, the ability to handle such large numbers of side-chains eases the burden of predicting or arbitrarily picking the few side-chains that need to change their conformation upon ligand binding. Future improvements to the scoring function and the representation of receptor flexibility are likely to further increase success rates. In particular, we plan to incorporate a focused sampling of rotatable ligand bonds, and published propensities for particular side-chains to undergo conformational changes to simplify the search. The addition of fully flexible representation of all terminal hydrogen atoms on receptor side-chains will also increase the accuracy of the scoring function. Finally, the addition of new motion descriptors for including local backbone motions to alter the Cα-Cβ bond orientation, and global motions to model loop and domain motions will increase the range of therapeutic targets for which this software can be used successfully. The software is freely available under an Open Source license at http://adfr.scripps.edu/ along with all data needed to reproduce the calculations presented in this paper.

## Supporting Information

S1 Text Genetic Algorithm Implementation (Additional Details). (DOCX)

S2 Text RMSD. (DOCX)

S3 Text Structure preparation. (DOCX)

S1 Table Cα deviations in the CDK2 data set. (DOCX)

S2 Table Astex Diverse Set and CDK2 datasets. (DOCX)

S3 Table SEQ17 dataset. (DOCX)

S4 Table SEQ17 cross-docking results. (DOCX)

S5 Table CDK2 cross-docking results. (DOCX)

S6 Table CDK2 docking success rates for *AutoDock Vina* with higher exhaustiveness. (DOCX)

S7 Table *AutoDock Vina* execution time scaling for varying exhaustiveness and levels of flexibility. (DOCX)
