# Supplementary material for: AutoDockFR: Advances in Protein-Ligand Docking with Explicitly Specified Binding Site Flexibility
Source: PLoS Comput Biol. 2015 Dec 2;11(12):e1004586. doi: 10.1371/journal.pcbi.1004586 (PMC4667975; doi:10.1371/journal.pcbi.1004586)
Supplement: S1 Text — (DOCX) [file pcbi.1004586.s001.docx]

**S1 Text:** Genetic Algorithm Implementation (Additional Details)

*Mutation*

The probability of genes to be selected for mutation across a generation is a user-specified parameter. When an individual is selected for mutation, the mutation operator is applied on a randomly picked subset of genes. The mutation operator of the orientation gene modifies the ligand quaternion values using a Gaussian distribution and renormalizes the quaternion. Ligand torsion genes are mutated using a Gaussian distribution centered on the current angle values, using a cyclic periodic boundary condition. The translation gene is mutated using a Gaussian distribution centered on the current values. The receptor side-chain genes are mutated using the soft rotamer library (1) as follows. A random number of receptor side-chains *N* to be mutated is obtained from a Gaussian distribution with an average of 2 and a standard deviation of 0.5; *N* side-chains are selected from the set of flexible side-chains and for each of them, a random conformation is generated by the Soft Rotamer operator (described in Methods).

*Crossover*

A GA crossover operator splits the genomes of two individuals in one or more locations and re-combines the pieces to create offspring. Crossover operations always occur at gene boundaries, e.g. the translation gene can only be exchanged as a whole (x, y, and z) between individuals. In the case of a rigid receptor, *ADFR* uses single point crossovers. When flexible side-chains are considered, two-point crossover is used with one crossover point in the ligand genes and one in the receptor genes.

*Local Search*

Solis-Wets local search method is used to minimize the energy of individuals. When an individual is selected for minimization, the algorithm randomly picks a set of genes, applies a small perturbation, and evaluates the fitness of the resulting individual. If the fitness improves, the algorithm keeps exploring in the same search space direction, increasing the perturbation magnitude upon successful iterations. If the fitness does not improve for a user specified number of consecutive attempts, the minimization stops.
