# Supplementary material for: AutoDockFR: Advances in Protein-Ligand Docking with Explicitly Specified Binding Site Flexibility
Source: PLoS Comput Biol. 2015 Dec 2;11(12):e1004586. doi: 10.1371/journal.pcbi.1004586 (PMC4667975; doi:10.1371/journal.pcbi.1004586)
Supplement: S2 Text — (DOCX) [file pcbi.1004586.s002.docx]

**S2 Text:** RMSD All RMSD values reported in this paper as well as RMSD used for clustering solutions in *ADFR* are computed using the Hungarian matching algorithm for pairing up atoms between the 2 binding modes. The Hungarian matching method is a combinatorial optimization algorithm that solves the assignment problem in polynomial time (1). Given a matrix representing the costs of each of *N* workers to perform any of *N* jobs, the goal is to assign jobs to workers in a way that minimizes the total cost knowing that each worker can perform only one job and each job can be assigned to only one worker. We use the open source Python implementation of the Hungarian matching algorithm (http://software.clapper.org/munkres/). We want to assign atoms of a given type in one pose to atoms of the same type in the other pose. Hence, we can solve the assignment problem for each atom type independently. The cost matrix we use to minimize the cost of the assignment contains the square of the distances between atoms of the same type. The cost of the produced match is the sum of the square of the distances between matched atoms. Hence, dividing the cost by the number of pairs and taking the square root of this number yields the RMSD of the match minimizing the cost.

REFERENCES

1. Kuhn HW. The Hungarian method for the assignment problem. Naval Research Logistics Quarterly. 1955;2(1-2):83-97.
