# Supplementary material for: AutoDockFR: Advances in Protein-Ligand Docking with Explicitly Specified Binding Site Flexibility
Source: PLoS Comput Biol. 2015 Dec 2;11(12):e1004586. doi: 10.1371/journal.pcbi.1004586 (PMC4667975; doi:10.1371/journal.pcbi.1004586)
Supplement: S3 Text — (DOCX) [file pcbi.1004586.s003.docx]

**S3 Text: Structure preparation.**  1) Astex Diverse Set: Structures were prepared for *AutoDock* and *ADFR* by converting receptor and ligand files from the original Mol2 format to the PDBQT format using the *prepare_receptor4.py* and the *prepareligand4.py* scripts from the *AutoDockTools* suite (1) respectively. 2) SEQ17: The receptor structures were prepared by adding hydrogen atoms, flipping side-chains and protonating Asn, Gln and His using *Reduce* (2) and by converting to PDBQT format using the *prepare_receptor4.py* script from *AutoDockTools* suite (1). The ligands were prepared by adding hydrogen atoms using OpenBabel (3) and converted to the PDBQT format using *prepare_ligand4.py* script from *AutoDockTools* suite (1). 3) CDK2: Dr. Daniel Santiago provided the CDK2 structures prepared for *AutoDock* using the *Maestro* software suite (Schrödinger Release 2014-4: Maestro, version 10.0, Schrödinger, LLC, New York, NY, 2014).

REFERENCES

1. Morris GM, Huey R, Lindstrom W, Sanner MF, Belew RK, Goodsell DS, et al. AutoDock4 and AutoDockTools4: Automated docking with selective receptor flexibility. Journal of computational chemistry. 2009;30(16):2785-91.

2. Word JM, Lovell SC, Richardson JS, Richardson DC. Asparagine and glutamine: using hydrogen atom contacts in the choice of side-chain amide orientation. J Mol Biol. 1999;285(4):1735-47.

3. O'Boyle NM, Banck M, James CA, Morley C, Vandermeersch T, Hutchison GR. Open Babel: An open chemical toolbox. J Cheminformatics. 2011;3.
