# Supplementary material for: AutoDockFR: Advances in Protein-Ligand Docking with Explicitly Specified Binding Site Flexibility
Source: PLoS Comput Biol. 2015 Dec 2;11(12):e1004586. doi: 10.1371/journal.pcbi.1004586 (PMC4667975; doi:10.1371/journal.pcbi.1004586)
Supplement: S1 Table — (DOCX) [file pcbi.1004586.s004.docx]

| Cα distances | ILE  10 | VAL  18 | LYS  33 | VAL  64 | PHE  80 | PHE  82 | GLN  85 | ASP  86 | LYS  89 | ASN  132 | LEU  134 | ASP  145 |
| --- | --- | --- | --- | --- | --- | --- | --- | --- | --- | --- | --- | --- |
| 1.1 | 9 | 4 | 0 | 0 | 2 | 0 | 2 | 3 | 0 | 0 | 3 | 1 |
| 1.2 | 4 | 2 | 0 | 0 | 5 | 0 | 0 | 1 | 3 | 1 | 1 | 3 |
| 1.3 | 4 | 4 | 0 | 0 | 2 | 0 | 2 | 1 | 2 | 0 | 0 | 2 |
| 1.4 | 4 | 2 | 2 | 0 | 1 | 0 | 0 | 0 | 1 | 0 | 0 | 1 |
| 1.5 | 2 | 2 | 0 | 0 | 1 | 0 | 0 | 0 | 0 | 0 | 0 | 1 |
| 1.6 | 3 | 0 | 3 | 0 | 0 | 0 | 0 | 0 | 0 | 0 | 0 | 5 |
| 1.7 | 4 | 1 | 1 | 0 | 0 | 0 | 0 | 0 | 0 | 0 | 0 | 4 |
| 1.8 | 0 | 1 | 1 | 0 | 0 | 0 | 0 | 0 | 0 | 0 | 0 | 3 |
| 1.9 | 0 | 1 | 1 | 0 | 0 | 0 | 0 | 0 | 0 | 0 | 0 | 1 |
| 2.0 | 1 | 0 | 1 | 0 | 0 | 0 | 0 | 0 | 0 | 0 | 0 | 0 |
| 2.1 | 0 | 1 | 1 | 0 | 0 | 0 | 0 | 0 | 0 | 0 | 0 | 0 |
| AVG | 1.05 | 0.85 | 0.58 | 0.31 | 0.44 | 0.28 | 0.50 | 0.43 | 0.50 | 0.53 | 0.33 | 0.83 |
| MAX | 1.96 | 2.01 | 2.01 | 0.99 | 1.49 | 0.87 | 1.29 | 1.3 | 1.37 | 1.1 | 1.15 | 1.82 |

**S1 Table:** Cα deviations in the CDK2 data set. For each flexible side-chain, this table provides the number of complexes (out of 52) for which the distance between the Cα atom in the *apo* and the *holo* structures is larger than 1.0Å. A significant number of amino acids for which side-chains are made flexible in docking calculations have a relatively large deviation in the Cα position, up to 2.01Å.
