# Supplementary material for: AutoDockFR: Advances in Protein-Ligand Docking with Explicitly Specified Binding Site Flexibility
Source: PLoS Comput Biol. 2015 Dec 2;11(12):e1004586. doi: 10.1371/journal.pcbi.1004586 (PMC4667975; doi:10.1371/journal.pcbi.1004586)
Supplement: S2 Table — (DOCX) [file pcbi.1004586.s005.docx]

| **A. Astex Diverse Set:** | | | | | | | | | | | | |
| --- | --- | --- | --- | --- | --- | --- | --- | --- | --- | --- | --- | --- |
| 1hp0 | 1l7f | 1n46 | 1pmn | 1r55 | 1t46 | 1u1c | 1v0p | 1w2g | 1y6b | 1ywr | 1hq2 | 1lpz |
| 1mzc | 1nav | 1q1g | 1sj0 | 1u4d | 1v48 | 1x8x | 1z95 | 1ke5 | 1lrh | 1n1m | 1of1 | 1oyt |
| 1q41 | 1r9o | 1tow | 1v4s | 1yqy | 1gpk | 1hwi | 1m2z | 1of6 | 1q4g | 1s19 | 1sqn | 1tt1 |
| 1unl | 1yv3 | 1hnn | 1hww | 1jje | 1l2s | 1opk | 1p62 | 1r1h | 1s3v | 1t40 | 1tz8 | 1uou |
| 1xoz | 2bsm | 1ia1 | 1j3j | 1t9b | 1kzk | 1k3u | 1vcj | 1ygc |  |  |  |  |
| **B. CDK2 data set:** | | | | | | | | | | | | |
| ***Apo*:** | | | | | | | | | | | | |
| 4ek3 |  |  |  |  |  |  |  |  |  |  |  |  |
| ***Holo*:** | | | | | | | | | | | | |
| 1h1p | 1h1q | 1h1r | 1h1s | 1jvp | 1pye | 1vyw | 1y8y | 1ykr | 2a4l | 2b52 | 2b53 | 2b55 |
| 2bkz | 2bpm | 2btr | 2bts | 2c6i | 2cch | 2duv | 2exm | 2fvd | 2g9x | 2j9m | 2r3f | 2r3i |
| 2r3q | 2uzo | 2v0d | 2w05 | 2w17 | 2wih | 3ddq | 3ezr | 3ezv | 4ek4 | 4ek5 | 4ek6 | 4ek8 |
| 4fkg | 4fki | 4fkj | 4fkl | 4fko | 4fkp | 4fkq | 4fkr | 4fks | 4fkt | 4fku | 4fkv | 4fkw |

**S2 Table:** Astex Diverse Set and CDK2 datasets. A) List of 85 PDB IDs from the Astex Diverse Set used to compare *ADFR* with *AutoDock*. B) CDK2 dataset used in the cross-docking study with flexible side-chains. The set includes one *apo* structure and 52 *holo* structures.
