# Supplementary material for: AutoDockFR: Advances in Protein-Ligand Docking with Explicitly Specified Binding Site Flexibility
Source: PLoS Comput Biol. 2015 Dec 2;11(12):e1004586. doi: 10.1371/journal.pcbi.1004586 (PMC4667975; doi:10.1371/journal.pcbi.1004586)
Supplement: S3 Table — (DOCX) [file pcbi.1004586.s006.docx]

| ***holo*** | ***apo*** | **Receptor** | **Ligand** | **Flexible side chains** |
| --- | --- | --- | --- | --- |
| 1it8 | 1iq8 | Archaeosine trna-guanine transglycosylase. | 2-amino-4-oxo-4,7-dihydro-3h-pyrrolo[2,3- d]pyrimidine-5-carbonitrile | Chain A: ASP95, SER98, ASP130, GLN169, VAL198, PHE229 |
| 1k4h | 1pud | Trna-guanine-transglycosylase | 2,6-diamino-8-propylsulfanylmethyl-3h-quinazoline- 4-one | Chain A: LEU68, ASN70, ASP102, TYR106, ASP156, CYS158, GLN203, MET260, ASP280 |
| 1gx9 | 1bsq | Beta-lactoglobulin | Retinoic acid | Chain A: LEU39, VAL41, LYS60, GLU62, LYS69, ILE71, LEU87, VAL92, PHE105, MET107, GLN120 |
| 1qkj | 2bgt | Beta-glucosyltransferase | Uridine-5'-diphosphate | Chain A: SER189, ARG191, ARG195, PHE213, ILE238, MET240, VAL243, ARG269, GLU272 |
| 2h8h | 1fmk | Proto-oncogene tyrosine-protein kinase Src | N-(5-chloro-1,3-benzodioxol-4-yl)-7-[2-(4- methylpiperazin-1-yl)ethoxy]-5-(tetrahydro- 2h-pyran-4-yloxy)quinazolin-4-amine | Chain A: LEU273, VAL281, LYS295, THR338, TYR340, ASN391, LEU393, ASP404 |
| 1lnm | 1kxo | Diga16 | digitoxigenin | Chain A: GLN28, HIS35, TYR39, TYR47, ARG58, HIS86, TYR88, PHE99, PHE114, LEU127, TRP129, LEU131 |
| 1z6p | 2gpn | Glycogen phosphorylase, muscle form | 4-{2-[(3-nitrobenzoyl)amino]phenoxy}phthalic acid | Chain A: TRP67, ILE68, GLN71, GLN72, ARG81, TYR155, ARG193, ARG310 |
| 3jrx | 2hjw | Acetyl-coa carboxylase 2 | soraphen a | Chain A: VAL273, LYS274, ARG277, SER278, ARG281, GLU593, MET594, ASN599, VAL648, GLU671, TRP681, PHE704 |
| 3erk | 1erk | Extracellular regulated kinase 2 | 4-(4-fluorophenyl)-1-(4-piperidinyl)-5-(2- amino-4-pyrimidinyl)-imidazole | Chain A: VAL37, LYS52, ILE82, ILE101, GLN103, ASP109, LYS112, LEU154 |
| 1ikg | 3pte | D-alanyl-d-alanine carboxypeptidase | Glycyl-l-alpha-amino-epsilon-pimelyl-d-alanyl- d-alanine | Chain A: SER62, LYS65, PHE120, THR123, TYR159, ASN161, LEU214, TRP233, ARG285, HIS298, THR299, THR301, GLN303, SER326 |
| 1rbp | 1brq | Plasma retinol-binding protein precursor | Retinol | Chain A: PHE36, LEU37, VAL61, LEU63, MET73, MET88, GLN98, HIS104 |
| 1aq1 | 1hcl | Cyclin-dependent protein kinase 2 | Staurosporine | Chain A: ILE10, VAL18, LYS33, PHE82, ASP86, LYS89, GLN131, LEU134, ASP145 |
| 1c1h | 1doz | Ferrochelatase | N-methylmesoporphyrin | Chain A: TYR13, TYR26, ILE29, ARG30, ARG31, PHE120, HIS183, LEU185, LYS188, TRP230, LEU263, GLU264 |
| 1br5 | 1rtc | Protein (ricin) | Neopterin | Chain A: TYR80, PHE93, TYR123, ILE172, SER176, GLU177, ARG180 |
| 1yxt | 1xqz | Proto-oncogene serine/threonine-protein kinase Pim-1 | phosphoaminophosphonic acid-adenylate ester | Chain A: PHE49, VAL52, LYS67, ILE104, ARG122, ASP167, LYS169, ASN172, LEU174, ILE185, ASP186 |
| 1zg3 | 1zhf | Isoflavanone 4'-O-methyltransferase | (2S,3R)-2,7-dihydroxy-3-(4-hydroxyphenyl)- 2,3-dihydro-4h-chromen-4-one | Chain A: ILE122, SER131, MET174, PHE175, TYR318, MET322, MET325, PHE326 |
| 2a9k | 2a78 | Ras-related protein Ral-A | nicotinamide-adenine-dinucleotide | Chain B: ASN87, ARG91, ARG128, ASP130, ASP131, TYR134, ARG167, GLU169, SER174, GLN182, PHE183, ARG186, GLU214 |

**S3 Table:** SEQ17 dataset. The set includes 17 *apo-holo* structures. The names of the receptor-ligand pairs along with the receptor side-chains made flexible are reported.
