# Supplementary material for: AutoDockFR: Advances in Protein-Ligand Docking with Explicitly Specified Binding Site Flexibility
Source: PLoS Comput Biol. 2015 Dec 2;11(12):e1004586. doi: 10.1371/journal.pcbi.1004586 (PMC4667975; doi:10.1371/journal.pcbi.1004586)
Supplement: S4 Table — (DOCX) [file pcbi.1004586.s007.docx]

| **Systems** | | **ADFR** | | | | **Vina** | | | |
| --- | --- | --- | --- | --- | --- | --- | --- | --- | --- |
|  |  | **RCD** | | **FCD** | | **RCD** | | **FCD** | |
| ***holo*** | ***apo*** | **RMSD** | **Rank** | **RMSD** | **Rank** | **RMSD** | **Rank** | **RMSD** | **Rank** |
| 1it8 | 1iq8 | 1.26 | 1 | 0.64 | 1 | 12.77 |  | 0.58 | 1 |
| 2h8h | 1fmk | 6.9 |  | 1.36 | 1 | 9.71 |  | 4.96 |  |
| 1k4h | 1pud | 6.26 |  | 2.06 | 1 | 4.15 |  | 0.88 | 1 |
| 1gx9 | 1bsq |  |  | 2.13 | 1 | 12.859 |  | 5.99 |  |
| 3jrx | 2hjw | 7.97 |  | 2.26 | 1 | 5.44 |  | 0.64 | 1 |
| 1qkj | 2bgt | 3.51 |  | 3.52 | 3 (1.85) | 7.75 |  | 7.79 |  |
| 1ikg | 3pte | 6.94 |  | 3.46 | 14 (1.29) | 3.1 | 3 (2.27) | 2.45 | 1 |
| 3erk | 1erk | 8.25 |  | 3.66 | 14 (0.77) | 4.23 |  | 6.93 |  |
| 1rbp | 1brq | 3.48 |  | 3.72 |  | 3.44 |  | 3.11 | 6 (1.55) |
| 1zg3 | 1zhf | 8.03 |  | 3.92 |  | 3.64 |  | 3.38 |  |
| 1aq1 | 1hcl | 7.46 |  | 3.97 | 2 (2.26) | 6.76 |  | 4.1 |  |
| 1z6p | 2gpn | 4.25 | 9 (1.86) | 4.28 | 2 (2.04) | 6.42 |  | 5.72 |  |
| 2a9k | 2a78 | 5.37 |  | 4.87 |  | 2.82 |  | 5.71 |  |
| 1yxt | 1xqz | 5.83 |  | 5.15 |  | 6.1 |  | 7.22 |  |
| 1br5 | 1rtc | 8.89 |  | 5.28 |  | 10.06 |  | 8.85 |  |
| 1c1h | 1doz | 5.62 |  | 5.41 | 14 (2.42) | 5.74 |  | 7.08 |  |
| 1lnm | 1kxo | 7.52 |  | 8.01 | 3 (2.28) | 11.27 |  | 7.88 | 4 (0.98) |

**S4 Table:** SEQ17 cross-docking results. The RMSD values of the best-scoring solutions are reported for *ADFR* and *AutoDock Vina* for both rigid cross-docking (RCD) and flexible cross-docking (FCD). In the case of false positive results (i.e. lowest energy solution has RMSD > 2.5Å) the rank and RMSD of the lowest energy correct solution is also reported.
