# Supplementary material for: AutoDockFR: Advances in Protein-Ligand Docking with Explicitly Specified Binding Site Flexibility
Source: PLoS Comput Biol. 2015 Dec 2;11(12):e1004586. doi: 10.1371/journal.pcbi.1004586 (PMC4667975; doi:10.1371/journal.pcbi.1004586)
Supplement: S5 Table — (DOCX) [file pcbi.1004586.s008.docx]

**S5 Table**: CDK2 cross-docking results. The RMSD values of the best-scoring solutions are reported for *ADFR* and *AutoDock Vina* for both rigid cross-docking (RCD) and flexible cross-dockings with 4, 10 and 12 flexible side-chains (FC4, FC10, FC12). In the case of false positive results (i.e. lowest energy solution has RMSD > 2.5Å) the rank and RMSD of the lowest energy correct solution is also reported.

| **Systems** | **ADFR** | | | | | | | | **Vina** | | | | | | | |
| --- | --- | --- | --- | --- | --- | --- | --- | --- | --- | --- | --- | --- | --- | --- | --- | --- |
|  | **RCD** | | **FC4** | | **FC10** | | **FC12** | | **RCD** | | **FC4** | | **FC10** | | **FC12** | |
|  | **RMSD** | **Rank** | **RMSD** | **Rank** | **RMSD** | **Rank** | **RMSD** | **Rank** | **RMSD** | **Rank** | **RMSD** | **Rank** | **RMSD** | **Rank** | **RMSD** | **Rank** |
| 1YKR | 5.96 |  | 5.97 |  | 0.27 | 1 | 0.34 | 1 | 7.85 |  | 5.99 |  | 0.77 | 1 | 0.84 | 1 |
| 2BTS | 8.48 |  | 1.57 | 1 | 0.48 | 1 | 0.37 | 1 | 8.55 |  | 8.54 |  | 3.17 |  | 8.59 |  |
| 4FKV | 6.46 |  | 2.54 |  | 1.14 | 1 | 0.78 | 1 | 3.82 |  | 3.12 |  | 5.74 | 2 (2.33) | 5.75 | 2 (2.41) |
| 4EK5 | 10.36 | 2 (0.81) | 0.60 | 1 | 9.91 | 2 (1.05) | 0.79 | 1 | 8.06 |  | 1.66 | 1 | 6.38 | 3 (1.65) | 9.08 | 2 (1.62) |
| 4FKS | 11.53 |  | 2.12 | 1 | 1.61 | 1 | 0.82 | 1 | 7.73 |  | 7.7 | 2 (2.31) | 1.88 | 1 | 1.74 | 1 |
| 4FKP | 6.13 |  | 2.47 | 1 | 0.86 | 1 | 0.90 | 1 | 6.12 |  | 2.46 | 1 | 1.02 | 1 | 0.94 | 1 |
| 4FKR | 1.65 | 1 | 2.37 | 1 | 1.32 | 1 | 0.92 | 1 | 4.57 |  | 2.1 | 1 | 1.04 | 1 | 2.06 | 1 |
| 2W17 | 5.96 |  | 4.38 |  | 3.49 | 3 (0.83) | 0.95 | 1 | 4.77 |  | 4.61 |  | 0.98 | 1 | 0.95 | 1 |
| 4FKG | 0.78 | 1 | 0.47 | 1 | 0.92 | 1 | 1.01 | 1 | 7.9 |  | 0.89 | 1 | 0.82 | 1 | 0.84 | 1 |
| 1Y8Y | 1.67 | 1 | 1.74 | 1 | 0.94 | 1 | 1.16 | 1 | 6.39 |  | 6.51 | 7 (1.92) | 6.51 | 4 (1.85) | 6.84 | 5 (2.38) |
| 2A4L | 10.23 | 9 (1.80) | 2.07 | 1 | 6.12 | 2 (1.36) | 1.18 | 1 | 3.01 |  | 1.66 | 1 | 1.73 | 1 | 6.06 | 3 (1.69) |
| 4FKQ | 14.73 | 5 (1.84) | 1.46 | 1 | 1.41 | 1 | 1.33 | 1 | 7.07 |  | 3.15 |  | 1.72 | 1 | 1.73 | 1 |
| 2W05 | 5.88 |  | 3.45 |  | 3.28 | 2 (1.48) | 1.33 | 1 | 7.59 |  | 5.98 |  | 6.02 |  | 1.28 | 1 |
| 2R3I | 1.90 | 1 | 1.24 | 1 | 1.57 | 1 | 1.49 | 1 | 1.57 | 1 | 2.72 | 6 (1.5) | 3.62 | 9 (1.83) | 2.76 |  |
| 4FKO | 2.20 | 1 | 6.47 | 2 (1.96) | 1.28 | 1 | 1.55 | 1 | 2.76 |  | 3.76 |  | 1.23 | 1 | 1.2 | 1 |
| 2R3Q | 2.04 | 1 | 1.22 | 1 | 1.97 | 1 | 1.58 | 1 | 1.32 | 1 | 4.67 | 2 (1.23) | 5.28 | 5 (1.86) | 5.32 |  |
| 1H1S | 5.32 | 2 (2.14) | 5.61 | 3 (2.40) | 1.59 | 1 | 1.62 | 1 | 6.64 |  | 6.31 |  | 6.37 | 4 (2.10) | 6.31 | 4 (2.04) |
| 4FKT | 11.06 |  | 2.57 | 3 (2.43) | 2.65 | 2 (2.25) | 1.69 | 1 | 9.02 |  | 9.18 | 7 (2.16) | 9.17 |  | 9.13 | 3 (2.33) |
| 2G9X | 4.98 |  | 5.96 |  | 1.99 | 1 | 1.70 | 1 | 7.11 |  | 7.36 |  | 5.86 | 8 (2.35) | 7.18 |  |
| 4EK8 | 8.94 |  | 1.99 | 1 | 2.10 | 1 | 2.07 | 1 | 5.35 |  | 5.32 | 2 (1.13) | 4.67 |  | 1.24 | 1 |
| 4FKU | 6.29 |  | 5.14 | 3 (1.87) | 1.14 | 1 | 2.15 | 1 | 6.68 |  | 6.48 | 2 (2.19) | 6.67 | 2 (2.12) | 6.51 | 3 (1.74) |
| 4FKW | 8.24 |  | 2.06 | 1 | 1.33 | 1 | 2.19 | 1 | 5.76 |  | 5.41 |  | 7.24 | 7 (1.5) | 5.38 | 9 (1.85) |
| 2BKZ | 2.53 |  | 2.52 |  | 2.45 | 1 | 2.22 | 1 | 2.88 |  | 2.82 |  | 3.55 |  | 4.96 |  |
| 2B53 | 2.23 | 1 | 5.70 | 2 (2.22) | 5.61 | 8 (1.99) | 2.89 | 19 (1.95) | 5.69 |  | 2.3 | 1 | 2.1 | 1 | 2.22 | 1 |
| 2R3F | 1.69 | 1 | 1.72 | 1 | 1.88 | 1 | 3.07 | 2 (1.65) | 1.66 | 1 | 3.04 | 4 (1.55) | 4.53 | 7 (1.44) | 4.34 | 7 (1.49) |
| 1H1R | 1.67 | 1 | 5.15 | 2 (2.05) | 5.06 | 4 (2.13) | 3.31 | 3 (2.06) | 5.75 |  | 5.49 | 8 (2.31) | 5.58 |  | 6.12 |  |
| 2B52 | 3.81 | 2 (2.04) | 3.61 | 2 (1.97) | 3.57 | 2 (1.84) | 3.67 | 2 (1.74) | 6.35 |  | 1.88 | 1 | 1.85 | 1 | 1.91 | 1 |
| 2B55 | 5.38 | 4 (1.92) | 4.12 | 2 (1.07) | 4.08 | 2 (1.31) | 4.07 | 2 (1.46) | 5.97 |  | 1.28 | 1 | 4.23 | 2 (1.24) | 1.36 | 1 |
| 4FKL | 13.47 |  | 4.09 | 3 (0.96) | 3.73 |  | 4.32 |  | 4.26 |  | 3.83 |  | 3.89 |  | 3.86 | 3 (2.27) |
| 1PYE | 3.08 |  | 3.29 | 5 (1.13) | 4.35 |  | 4.46 |  | 5.52 |  | 3.42 |  | 3.53 | 4 (2.34) | 3.28 | 5 (2.08) |
| 2DUV | 4.33 |  | 4.31 | 3 (2.46) | 4.48 | 11 (1.75) | 4.58 | 14 (1.54) | 5.82 |  | 4.45 |  | 3 |  | 3.2 |  |
| 2UZO | 5.49 |  | 5.41 | 2 (1.89) | 5.41 |  | 4.78 |  | 3.65 |  | 2.49 | 1 | 1.82 | 1 | 2.33 | 1 |
| 1JVP | 4.48 | 2 (2.3) | 2.76 | 2 (1.33) | 0.98 | 1 | 4.86 | 2 (1.06) | 4.31 |  | 2.21 | 1 | 2.85 |  | 2.72 |  |
| 2J9M | 1.56 | 1 | 4.02 | 4 (1.34) | 5.10 | 6 (1.61) | 4.87 | 7 (1.3) | 5.4 |  | 5.51 |  | 5.58 |  | 5.52 | 5 (1.79) |
| 3EZR | 7.59 |  | 5.38 |  | 5.41 |  | 5.25 |  | 7.86 |  | 5.57 |  | 7.3 |  | 5.92 |  |
| 1H1Q | 5.40 | 3 (1.65) | 5.39 | 5 (1.83) | 7.30 | 7 (1.89) | 5.28 | 7 (1.96) | 6.35 |  | 5.28 | 5 (2.02) | 6.82 | 8 (2.36) | 6.83 |  |
| 3EZV | 7.03 |  | 5.37 |  | 5.24 |  | 5.40 |  | 5.86 |  | 5.67 |  | 5.9 |  | 5.92 |  |
| 3DDQ | 5.28 |  | 2.60 | 8 (1.65) | 6.08 | 3 (2.33) | 5.93 | 2 (2.4) | 6.13 | 6 (2.39) | 2.41 | 1 | 1.99 | 1 | 2.41 | 1 |
| 2BTR | 8.36 |  | 0.51 | 1 | 5.75 | 2 (2.32) | 6.10 | 2 (1.12) | 7.19 |  | 6.69 |  | 7.06 | 3 (0.28) | 7.11 |  |
| 1H1P | 5.56 |  | 5.30 |  | 5.73 |  | 6.20 |  | 7.92 |  | 2.77 |  | 7.03 |  | 7.32 |  |
| 2WIH | 7.02 |  | 2.13 | 1 | 6.96 | 4 (1.86) | 6.69 | 4 (1.29) | 5.76 |  | 3.77 |  | 3.35 | 2 (1.92) | 3.51 | 2 (1.66) |
| 2C6I | 7.75 |  | 2.35 | 1 | 0.18 | 1 | 7.38 | 2 (1.58) | 7.09 |  | 7.01 |  | 7.73 |  | 1.12 | 1 |
| 2BPM | 13.09 | 4 (1.68) | 5.62 | 4 (1.78) | 5.13 | 12 (1.94) | 7.42 | 18 (1.47) | 6.83 |  | 7.7 |  | 7.7 |  | 7.87 |  |
| 4FKJ | 14.26 | 3 (1.49) | 14.28 | 2 (1.47) | 5.03 | 7 (1.54) | 7.46 | 9 (1.38) | 7.41 |  | 7.96 |  | 6.63 |  | 10.32 |  |
| 2FVD | 8.34 |  | 8.44 |  | 7.91 |  | 7.82 |  | 7.32 |  | 7.18 |  | 7.1 |  | 7.07 |  |
| 2CCH | 8.83 |  | 8.61 |  | 7.59 | 17 (2.25) | 8.07 |  | 3.01 |  | 3.36 | 3 (2.19) | 7.02 |  | 3.58 | 7 (2.39) |
| 2EXM | 9.66 |  | 2.68 | 2 (2.50) | 5.27 | 5 (1.91) | 8.83 | 2 (2.29) | 5.14 | 5 (2.33) | 5.57 |  | 4.72 |  | 6.89 |  |
| 2V0D | 13.01 |  | 4.15 |  | 4.31 |  | 9.38 |  | 4.12 | 2 (2.35) | 3.98 | 7 (1.65) | 3.98 | 6 (2.07) | 3.98 | 5 (1.64) |
| 4EK6 | 14.00 | 2 (2.39) | 2.39 | 1 | 7.44 | 5 (2.27) | 9.84 | 5 (2.36) | 7.43 |  | 7.53 |  | 7.41 |  | 10.2 |  |
| 4EK4 | 0.41 | 1 | 10.56 | 2 (0.57) | 10.86 | 9 (0.69) | 10.21 | 7 (0.45) | 1.28 | 1 | 1.29 | 1 | 1.24 | 1 | 4.92 | 2 (1.23) |
| 1VYW | 14.00 | 6 (1.63) | 1.06 | 1 | 9.68 | 4 (1.62) | 10.22 | 4 (1.5) | 7.56 |  | 8.32 |  | 10.12 |  | 9.46 |  |
| 4FKI | 1.58 | 1 | 2.59 | 2 (2.12) | 4.79 | 8 (1.45) | 10.55 | 9 (2.12) | 7.42 |  | 8.71 | 9 (2.29) | 6.39 |  | 9.13 |  |
