# Supplementary material for: AutoDockFR: Advances in Protein-Ligand Docking with Explicitly Specified Binding Site Flexibility
Source: PLoS Comput Biol. 2015 Dec 2;11(12):e1004586. doi: 10.1371/journal.pcbi.1004586 (PMC4667975; doi:10.1371/journal.pcbi.1004586)
Supplement: S6 Table — (DOCX) [file pcbi.1004586.s009.docx]

| **Flexible Side Chains** | | **Solutions** | | ***AutoDock Vina* with higher exhaustiveness** | | | |
| --- | --- | --- | --- | --- | --- | --- | --- |
|  |  | **Rank 1** | **Rank < 10** | **1** | **<10** | **1** | **<10** |
| **# Active** | **# Rot. χ angles** | **Vina8** | | **Vina20** | | **Vina200** | |
| 0 | 0 | 4 | 5 | 4 | 7 | 4 | 7 |
| 4 | 10 | 6 | 13 | 6 | 13 | 4 | 12 |
| 10 | 22 | 13 | 24 | 10 | 27 | 13 | 27 |
| 12 | 27 | 12 | 23 | 10 | 23 | 11 | 27 |

**S6 Table:** CDK2 docking success rates for *AutoDock Vina* with higher exhaustiveness. The number of rotatable χ angles corresponding to 0, 4, 10 and 12 flexible side-chains are reported along with the number of correct docking solutions for *AutoDock Vina* with exhaustiveness values of 8, 20 and 200.
