# Supplementary material for: AutoDockFR: Advances in Protein-Ligand Docking with Explicitly Specified Binding Site Flexibility
Source: PLoS Comput Biol. 2015 Dec 2;11(12):e1004586. doi: 10.1371/journal.pcbi.1004586 (PMC4667975; doi:10.1371/journal.pcbi.1004586)
Supplement: S7 Table — (DOCX) [file pcbi.1004586.s010.docx]

| **# Flexible Side Chains** | **Vina8** | **Vina20** | **Vina200** |
| --- | --- | --- | --- |
| **0** | 1.00 | 2.46 | 23.61 |
| **4** | 7.22 | 16.53 | 163.06 |
| **10** | 34.00 | 97.67 | 949.21 |
| **12** | 61.83 | 164.51 | 1499.82 |

**S7 Table:** *AutoDock Vina* execution time scaling for varying exhaustiveness and levels of flexibility. The table provides *AutoDock Vina* execution times divided by the time needed to perform rigid docking with exhaustiveness 8. The first row provides a measure of time scaling as a function of exhaustiveness. Variations of columns describe time increase as a function of the amount of receptor flexibility.
